# Physics-Informed Bayesian learning of electrohydrodynamic polymer jet printing dynamics

**DOI:** 10.1038/s44172-023-00069-0

**Published:** 2023-04-29

**Authors:** Athanasios Oikonomou, Theodoros Loutas, Dixia Fan, Alysia Garmulewicz, George Nounesis, Santanu Chaudhuri, Filippos Tourlomousis

**Affiliations:** 1 Mechanical Engineering, University of Patras, Patras, Greece; 2 National Centre for Scientific Research Demokritos, Agia Paraskevi, Attica, Greece; 3 Superlabs AMKE, Marousi, Attica, Greece; 4 Westlake University, Hangzhou, China; 5 Faculty of Economics and Administration, University of Santiago, Santiago, Chile; 6 Civil, Materials, and Environmental Engineering Department, University of Illinois at Chicago, Chicago, IL USA; 7 Argonne National Laboratory, Lemont, IL USA; 8 Biological Lattice Industries Corp, Boston, MA USA

**Keywords:** Mechanical engineering, Information technology, Design, synthesis and processing, Chemical engineering

## Abstract

Calibration of highly dynamic multi-physics manufacturing processes such as electrohydrodynamics-based additive manufacturing (AM) technologies (E-jet printing) is still performed by labor-intensive trial-and-error practices. Such practices have hindered the broad adoption of these technologies, demanding a new paradigm of self-calibrating E-jet printing machines. Here we develop an end-to-end physics-informed Bayesian learning framework (GPJet) which can learn the jet process dynamics with minimum experimental cost. GPJet consists of three modules: the machine vision module, the physics-based modeling module, and the machine learning (ML) module. GPJet was tested on a virtual E-jet printing machine with in-process jet monitoring capabilities. Our results show that the Machine Vision module can extract high-fidelity jet features in real-time from video data using an automated parallelized computer vision workflow. The Machine Vision module, combined with the Physics-based modeling module, can also act as closed-loop sensory feedback to the Machine Learning module of high- and low-fidelity data. This work extends the application of intelligent AM machines to more complex working conditions while reducing cost and increasing computational efficiency.

## Abstract (web summary)

Athanasios Oikonomou and colleagues develop a robust and computationally efficient multi-fidelity modeling framework that can learn the jet dynamics of melt electrowriting from videos and physical models. This learning strategy paves way for the next generation of self-calibrating electrohydrodynamics-based additive manufacturing technologies.

## Introduction

The programmable assembly of functional inks in two- and three-dimensions using computer numerically controlled (CNC) machines coupled with printing technologies has revolutionized the design and fabrication of physical objects. Extrusion-based additive manufacturing (AM) technologies, often referred to as direct ink writing or 3D printing, are transforming fields such as healthcare, robotics, electronics, and sustainability^1, 2^. While the potential of 3D printing is celebrated very often in scientific journals and the media, there is a “secret” that practitioners and companies of 3D printing do not emphasize. This under-reported reality entails the extensive experimentation and manual labor required for tuning process parameters that are high in number and often inter-dependent, to achieve process stability and reproducible outcomes^3^. Every time a new material needs to be processed, or ambient conditions vary, practitioners follow trial and error approaches for printing process calibration. These calibration practices have led to the creation of experienced “super users” at the expense of an enormous degree of individual process engineering.

**Fig. 1:**
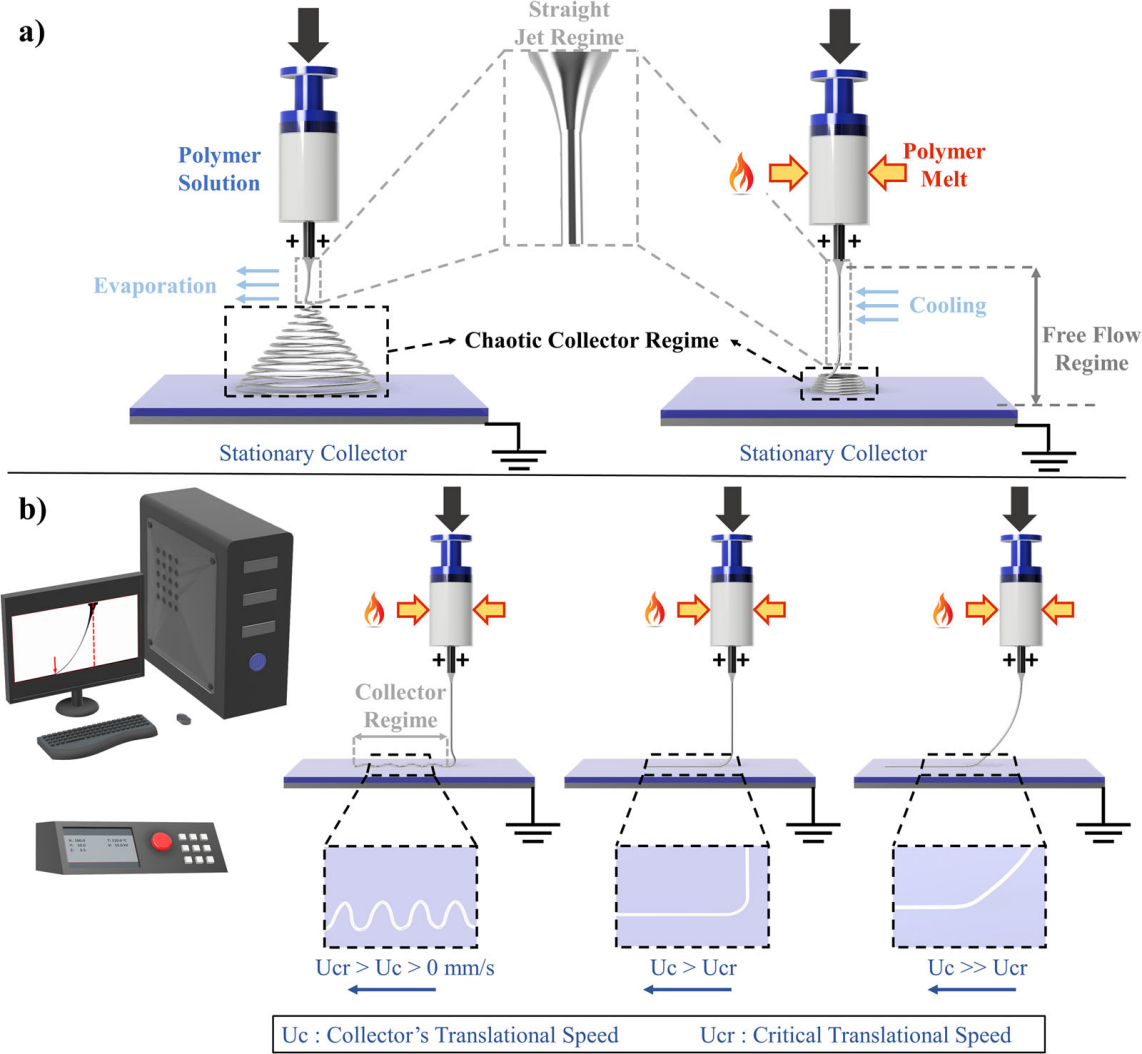
Electrohydrodynamic Jet Printing Process. **a** Solution electrospinning (SES) vs. melt electrospinning (MES). The main differentiating feature between the two processes is the extent of the jet instabilities that arise from the electrostatic forces acting at the polymer jet-air interface. For MES, the chaotic jet regime is limited close to the grounded collector plate due to the high viscosity and dielectric properties of the pure polymer melt (external heat (red flame) applied on the syringe barrel (red-yellow arrows)). **b** Melt electrowriting (MEW) and its operating principle that is based on the direct writing of melt electrospun fibers on a grounded collector plate that is mounted on a cartesian x-y robotic stage. Different fiber topographies can be achieved during printing by tuning the translational collector speed, ${U}_{c}$ [mm s^−1^] in each axis. Straight fiber topographies can be achieved at the critical collector speed ${U}_{{cr}}$ [mm s^−1^] with a jet deposition position right below the needle tip. At collector speeds considerably higher than the critical collector speed, a jet lag phenomenon is observed due to excess stretching of the viscoelastic jet.

Electro-hydrodynamics-based AM technologies, also known as E-jet printing technologies, are notable examples of extrusion-based AM technologies that have been facing such challenges due to their complex multi-physics and highly dynamic nature^4,5^. A wide variety of materials, also termed as inks, can be processed with E-jet printing technologies. Processable inks include homogenous solution (pure solvents or solubilized materials), suspensions (such as colloids of quantum dots, nanoparticles, insoluble material), melts (such as molten metal, wax, etc.), biomolecules (DNA, proteins, and bacteria) and polymers (solutions or melts). During E-jet printing, the ink is extruded through a charged needle tip towards a grounded collector. As soon as the electrostatic stresses overcome the polymer material’s viscoelastic and surface tension stresses, a cone-jet is formed in the free flow regime (Fig. 1a). An instabilities area, whose span size across the jet depends on the nature of the polymer (solution or melt), follows the cone-jet regime. Focusing on the polymer melt case, where the instabilities area is closer to the collector (Fig. 1a), a translational stage can be employed to write high-resolution fibers (Fig. 1b), a process known as melt electro-writing (MEW). With this capability, MEW has been established as an emerging high-resolution AM technology for fabricating architected biomaterial scaffolds, opening new tissue engineering avenues. MEW has undergone ten years of process optimization studies since its first inception in the literature^6,7^. Tunable fiber diameter and patterning fidelity are critical scaffold attributes for biological outcomes and efficacy. These can be optimized by tuning five inter-dependent user-controlled process parameters assuming stable ambient environmental conditions (temperature and humidity): (a) the applied voltage at the needle tip, (b) the extrusion volumetric flowrate, (c) the temperature at the syringe, (d) the collector speed, and (e) needle tip to collector distance. Considering the dynamic range of each process variable in combination with the highly sensitive spatial and time scales of the process in the micron range, one quickly realizes why it took 10 years for process optimization with the vast majority of these studies using one specific material i.e., polycaprolactone (PCL).

Earlier studies achieved printing fidelity with MEW using an approach based on intuition, i.e., manually selecting values for the critical process parameters, performing post-printing fidelity measurements, assessing trends and patterns in data, and selecting process parameter settings for follow-up experimentation. Later studies focused on understanding the previously identified printing regimes with respect to the physics and the dynamics of the process^8–10^. A recent study systematically approached the calibration process by exploring the parameter space using a Design-of-Experiments approach in a simple Cartesian grid defined by the number of independent process parameters^11^. In this study, computer vision was employed to image the jet in the free-flow regime as a function of various process parameter conditions in a high throughput manner^11^. The generated dataset was then assessed offline to identify high fidelity printability regimes^11^. However, selecting an exploration strategy implies picking a resolution without knowing the model function. To address that, the resolution is often chosen high, aiming for an exhaustive search to avoid inaccuracies. With the high dimensionality of the parameter space, this brute force data collection method quickly fails to explore the space efficiently and becomes prone to bias.

The challenges mentioned before, combined with the demand for increasingly complex and reproducible products, warrant a new paradigm for E-jet printing machines. In this paradigm, rigid machines calibrated by trial-and-error practices are replaced by “intelligent” autonomous machines capable of adapting and learning process dynamics with minimum experimental cost. Artificial intelligence and machine learning (ML) are transforming many areas of experimental science in this direction. However, advances in manufacturing science are mainly driven by expensive physics-based simulations that cannot resolve all scales and, more recently, by data-hungry neural networks trained offline with in-process monitoring datasets for defect detection and process performance prediction on various AM platform technologies^12^.

To address these challenges, we adopt an approach inspired by the operating principles behind autonomous materials experimentation platforms, also known as research robots^13–15^ and from the field of physics-informed machine learning^16,17^. Research robots demonstrate closed-loop control through online learning from prior experiments, planning and execution of new experiments. Physics-informed machine learning lays the foundations for integrating data with domain knowledge in the form of mathematical models to allow efficient simulations of highly multi-physics phenomena. The underlying framework of research robots provides a systematic data-driven approach for the identification of the best follow-up experiments to optimize unknown functions. The functions are approximated by Gaussian Process Regression (GPR), which is a robust statistical, nonparametric technique both for function approximation and uncertainty quantification^18,19^. During the Bayesian optimization loop, an acquisition function balances the utilization of experiments that explore the unknown function with experiments that exploit prior knowledge by considering the quantified uncertainty after each function approximation step^20^. Efficiency with respect to the utilization of experimental resources could be further improved by augmenting the surrogate model with prior domain knowledge following a multi-fidelity modeling approach^21–23^. The success of this approach has been documented in the field of computational science by using simple and potentially inaccurate models that carry a low computational cost to achieve predictive accuracy on a small set of high-fidelity observations obtained from accurate models that carry a high computational cost.

Automated materials experimentation systems driven by Bayesian optimization active learning frameworks have demonstrated remarkable performance in autonomously searching the vast synthesis-process-structure-property landscape resulting to the accelerated discovery of advanced materials for a wide variety of applications^20,24–28^ including AM^29,30^. However, the application of autonomic principles for the calibration of AM processes remains underexploited. In one study concerning E-jet printing of substrates with micron-scale topographical features, the authors demonstrated a research robot, whose planner is informed by an in-line nano-surface metrology tool and actively learns to tune the extrusion rate until it achieves a predefined topographical feature^31^. In another study about direct ink writing of paste materials, the authors demonstrated an autonomous 3D printer whose planner is informed by machine vision cameras and adaptively searches the space of four process parameters to print single struts with geometrical features that match user-defined specifications^32^.

In this paper, we employ principles from autonomous research robots to develop an end-to-end physics-informed probabilistic machine learning framework that sets the basis for the next generation of self-calibrating E-jet printing machines. Such a framework should allow both online extraction of jet features from in-process monitoring data and online robust modeling of process signature dynamics using the extracted data in the most computationally efficient way. Thus, we have followed a data-centric approach that leverages data of multiple fidelities from experiments and physics-domain knowledge, to demonstrate the utility of the framework both in an offline but also in an online process calibration scenario.

To accomplish that, we construct a virtual MEW machine using a previously published video dataset acquired by a conventional camera that performs in situ jet monitoring under various process conditions, and we demonstrate that our data-driven framework called GPJet is capable of:

- high-fidelity jet feature extraction in real-time from video data using a parallelized computer vision algorithmic workflow that is systematically profiled under various implementations,
- low-fidelity jet feature extraction from “cheap” physics-based models describing the evolution of the jet across the free-flow regime and the deposition dynamics of a gravity-driven viscous thread onto a moving surface known as the “fluid-mechanical sewing machine.”
- With these capabilities, we demonstrate that GPJet is a robust multi-fidelity modeling framework that can learn the process dynamics with minimum experimental cost as described by the required number of high-fidelity data.

Our results are supported by performance tests comparing offline and online calibration scenarios revealing that the online ML planner, based on an active learning approach that balances exploration and exploitation, can effectively learn the jet evolution in the free-flow regime much more efficiently when it is informed by physics and based on that to adaptively tune the translational speed of the collector for minimum jet lag distance. In that case, the ML planner follows a decision-making strategy revealing the universality of the fluid mechanical sewing machine model in predicting the deposition dynamics of any printing process of viscous jets no matter what the nature of the jet driving force is.

## Results

### GPJet: the physics-informed machine learning pipeline

**Fig. 2:**
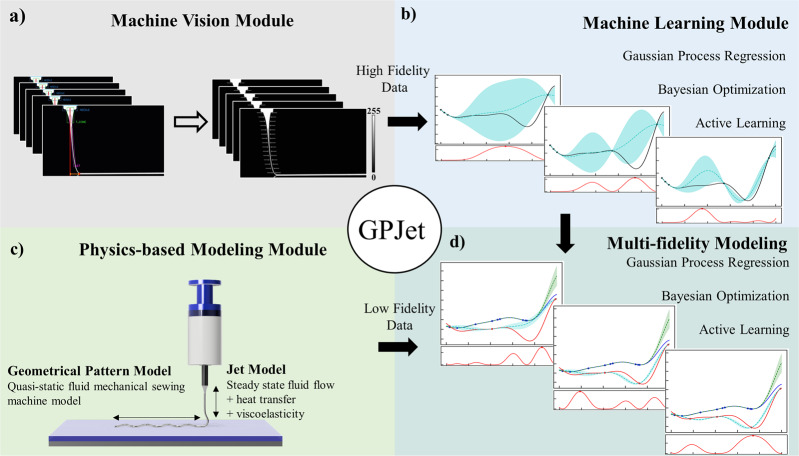
The GPJet Pipeline Framework. The Physics-informed Bayesian Machine Learning framework (GPJet) comprised by three different modules: **a** the Machine Vision module, which takes as an input timeseries video focusing on the polymer jet in the free flow regime and performs the extraction of high-fidelity jet features in real-time based on an automated image processing algorithmic workflow (denoted as “Jet Metrology” in Section Machine Vision Module) – the extracted jet features are denoted on jet profile images in grayscale (0–255) with the 0 value and the 255 value in the color bar representing the black background, and white segmented jet profile respectively, **b** the Machine Learning module and **c** the Physics-based Modeling Module, **d** the Multi-fidelity Modeling Module which takes as input high fidelity experimental data from the Machine Vision module and low fidelity modeling data from the Physics-based Modeling Module and performs a series of data-driven tasks to learn the jet dynamics. Filled contours (shading) represent uncertainty bounds (95% confidence intervals (CIs)) of the predictions.

To demonstrate the ability of learning the dynamics of E-Jet printing processes in a data-driven fashion, we employ a pipeline-based approach that is depicted in Fig. 2. The approach is composed of three modules, namely: the machine vision module, the physics-based modeling module, and the machine learning module. In GPJet, features that are representative of the printing process dynamics, are extracted by the machine vision module and the physics-based modeling module. In the context of this paper, high-fidelity observations are referred to the jet features extracted experimentally, and low-fidelity observations are referred to the same jet features as predicted by a low-cost numerical model that is a good approximation of the reality.

As a first step, jet features are engineered and extracted in real-time using an algorithmic computer vision workflow taking as an input time-series video data (see Methods for details). The Machine Vision module allows us to probe and measure the jet dynamics, a capability hereafter denoted as jet metrology. The jet metrology serves as a feature extraction step of high-fidelity observations corresponding to the jet radius profile (${R}_{j}$ [mm]) and the jet lag distance (${L}_{j}$ [mm]), which are then fed into the Machine Learning module that can perform various Bayesian-based batch and online learning tasks (see Methods for details). The Machine Learning module can be further informed by low-fidelity observations, a capability hereafter denoted as Multi-fidelity modeling. The low-fidelity observations are obtained by the Physics-based modeling module and correspond to the same engineered features that are extracted experimentally by the Machine Vision module (${R}_{j}$ [mm] and (${L}_{j}$ [mm]).

Collectively, the GPJet pipeline offers a range of unique capabilities ranging from real-time feature extraction using computer vision to physics-informed machine learning capabilities that aim to minimize experimental cost without sacrificing accuracy and robustness.

### Dataset

To demonstrate the utility and performance of the GPJet pipeline, we curated a dataset that emulates a virtual E-jet printing machine with a dynamic range of 12 user-controlled machine settings. The dataset is depicted in Table S1 and is created based on previously published time-series video data^10^. Specifically, the raw data is acquired by a conventional camera with 50 fps and a field of view spanning the area between the needle tip and the grounded collector of a melt electro-writing (MEW) system. A detailed explanation of the raw data, the preprocessing procedure derive the final curated dataset can be found in Supplementary Note. MEW constitutes an ideal testbed for demonstrating the capabilities and the flexibility of our GPJet framework. The highly dynamic nature of the process and the multiple user-controlled independent process parameters, pose several challenges that we demonstrate both in an offline and an online self-calibrating machine scenario.

### Learning jet dynamics from videos

**Fig. 3:**
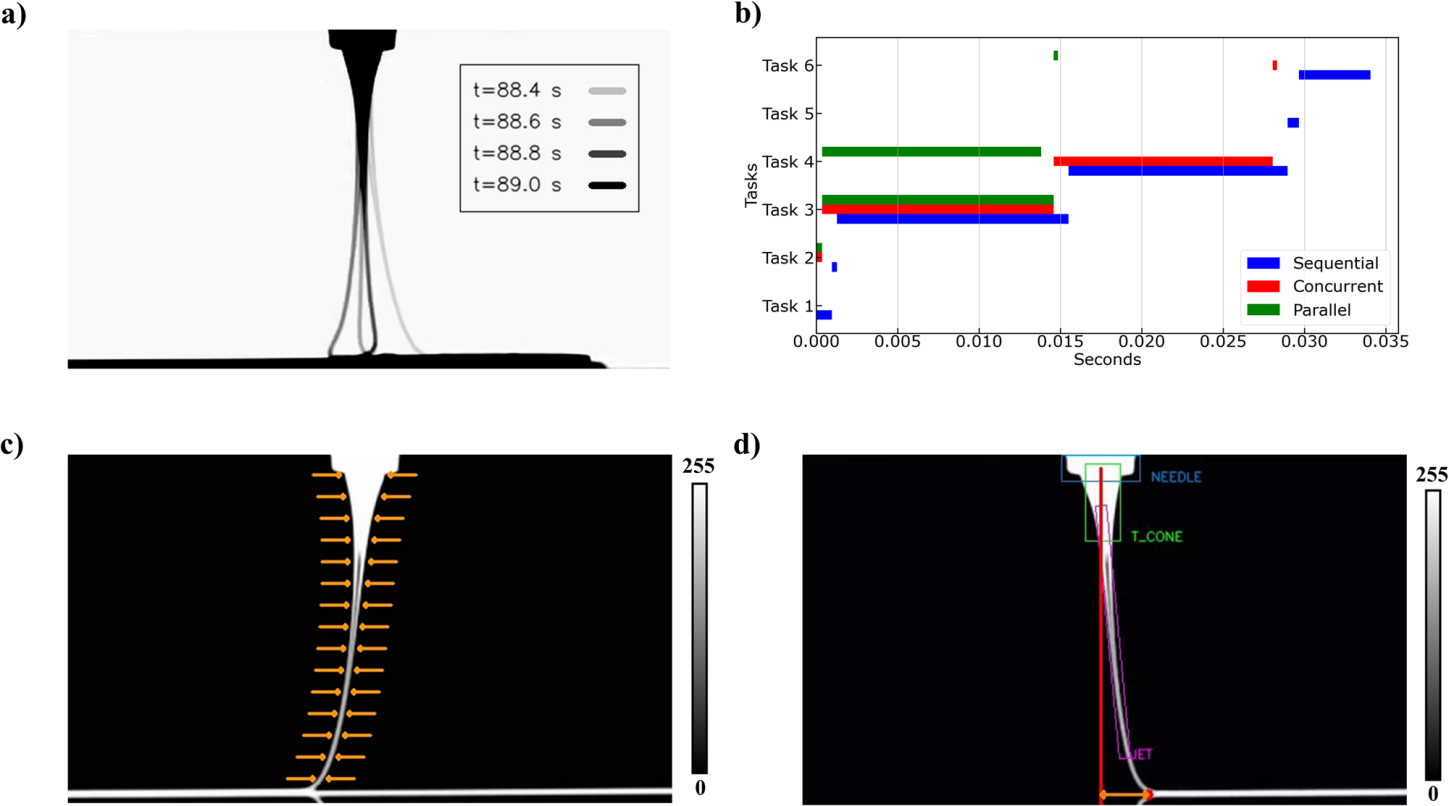
Machine Vision Module. **a** Process dynamics and its time scale represented by overlayed jet profile images of the jet profile at different time points. **b** Profiling experiments for different code implementations. **c** Edge-based feature extraction methodology (Task 3 in Fig. 3b), features are extracted along the jet in the positions denoted by the orange arrows on. **d** Object-based feature extraction methodology (Task 4 in Fig. 3b), the needle, the Taylor cone and the jet are enclosed by a light blue, a light green and a magenta box, respectively. The imaginary direct line from the center of the needle to the collector, the deposition point, and the jet lag distance are depicted with a red line, a red dot, and a two-ways orange arrow, respectively. The jet profile images in **c** and **d** are images in grayscale (0–255) with the 0 value and the 255 value in the color bar representing the black background, and white segmented jet profile, respectively.

As a first goal we set out to tackle the challenge of real-time process monitoring and jet metrology. To demonstrate the highly dynamic nature of the process, we plot overlaid video frames showing the jet hitting a stationary collector (Fig. 3a). We chose to plot frames with a time step equal to 0.2 s since the electrostatic nature of the process and the viscoelasticity of the molten jet cause instabilities of a smaller time scale (~0.02 s) and result in jet topologies that are indistinguishable with a naked eye. This number provided a starting point for setting a goal related to the computational efficiency of the machine vision module for real-time performance. Since the camera acquisition time was equal to 0.02 s (50 fps), we proceeded with the goal to maintain computational processing time equal or smaller than that.

To accomplish this, we started by dividing the computer vision workflow in specific algorithmic tasks and implemented a sequential code version. We continued by systematically profiling the code, identifying the computationally expensive tasks, and then gradually parallelizing the code to reduce computational processing time. This approach led to three different code implementations of the machine vision module: (a) the sequential, (b) the concurrent and the (c) parallel, with the last one achieving real-time performance. The results of the profiling experiments are shown in Fig. 3b, where all the tasks are plotted along with their respective processing time for the three different code implementations.

Specifically, the machine vision tasks per frame are the following:

Task 1: Read new video frame.

Task 2: Process the frame to reverse background color.

Task 3: Edge-based feature extraction and data storage.

Task 4: Object-based feature extraction and data storage.

Task 5: Show processed video output.

Task 6: Save video output.

Profiling the sequential code version reveals that an average time of 0.033 s. is needed to perform the whole machine vision workflow per frame with the most expensive task being the one that performs edge-based feature extraction across the jet length (Fig. 3c). To alleviate this source of computational cost, we employed a multi-threading strategy for the concurrent code version that led to a modest improvement of 0.005 s.

Multi-threading is implementing software to perform two or more tasks in a concurrent manner within the same application. Multi-threading employs multiple threads to perform each task with no limitation in the number of threads that can be used^10^. We learned that multithreading on one hand can reduce processing time of I/O bound tasks almost to zero, but on the other hand does not improve processing time of Central Processing Unit (CPU) bound tasks, such as Task 3 and Task 4, which are the most expensive.

To further reduce processing time, we augmented the concurrent version with a multi-processing strategy that led to the parallel code version. Multi-processing systems have multiple processors running at the same time. Therefore, different tasks of an application can be run in different processors in a parallel manner. This capability considerably accelerates program performance. The limitation of this strategy is related to the fact that the number of processes that can be employed must be less or equal to the number of processors (CPU cores) of the device^10^. Finally, by employing multi-threading for I/O bound tasks (Task 1, Task 5, and Task 6) and multi-processing for CPU bound tasks (Task 3, Task 4), we were able to achieve real-time process monitoring and jet metrology with processing time up to 0.014 s.

Instrumented with the capability to perform jet feature extraction in real-time, we then focused on quantifying process dynamics relevant features. With the edge-based feature extraction algorithm, which is described in detail in Learning Jet Dynamics from Videos & Physics under the Methods section, we were able to measure the jet diameter profile, the area of the whole jet, the angle between the vertical line that connects the nozzle tip with the collector, and different points across the length of the jet profile and finally the translational jet speed at different points across the length of the jet profile. The high content spatiotemporal results are plotted in Fig. S1 of the Supplementary Information demonstrating the breadth of information of the machine vision module and the fact that the jet point right above the collector undergoes a highly fluctuating behavior that will directly affect the printing quality.

**Fig. 4:**
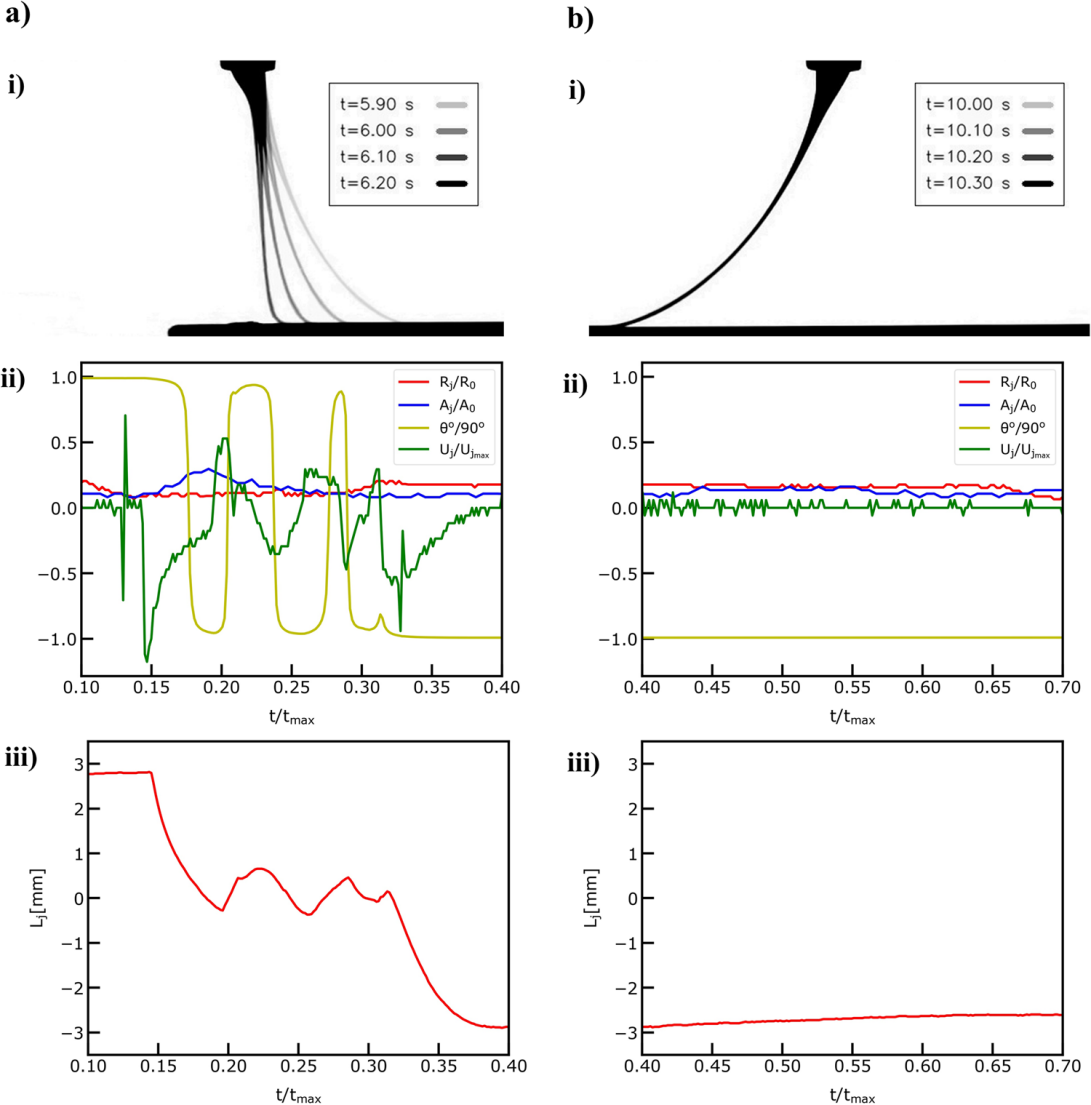
Jet Metrology with the Machine Vision Module. **a** The extracted features during the deceleration-acceleration phase of the printing process. **i** Overlayed video frames demonstrating the dynamics during the deceleration-acceleration phase and normalized jet length point of interest ($Z/{R}_{o}|{R}_{0}=17.5$) denoted with red color. **ii** Normalized jet radius (${R}_{j}/{R}_{o}\,$), Normalized jet area (${A}_{j}/{A}_{o}\,$), Normalized jet angles ($\theta /{90}^{o}$) and Normalized jet velocity (${U}_{j}/{U}_{{jmax}}$) at the denoted point of interest plotted against the normalized time ($t/{t}_{\max }\,$) during the deceleration-acceleration phase. **iii** Jet lag distance $({L}_{j})$ plotted against the normalized time ($t/{t}_{\max }\,$) during the deceleration-acceleration phase. **b** The extracted features during the steady speed phase pf the printing process**. i** Overlayed video frames demonstrating the dynamics during the steady speed phase. **ii** Normalized jet radius (${R}_{j}/{R}_{o}\,$), Normalized jet area (${A}_{j}/{A}_{o}\,$), Normalized jet angles ($\theta /{90}^{o}$) and Normalized jet velocity (${U}_{j}/{U}_{{jmax}}$) at the denoted point of interest plotted against the normalized time ($t/{t}_{\max }\,$) during the steady speed phase. **iii** Jet lag distance $({L}_{j})$ plotted against the normalized time ($t/{t}_{\max }\,$) during the steady speed phase.

We present the jet metrology results for two distinct phases during the printing process in Fig. 4ai-ii and Fig. 4bi-ii focusing on the jet point right above the collector, hereafter denoted as point of interest. With the object-based feature extraction algorithm which is described in detail in sub-section 4.1 under the Methods section, we were able to detect key objects in the field of view such the needle tip, the Taylor cone, which is defined as the jet area between the needle tip outlet and the jet point 2*Ro away from the needle tip, the remaining jet, and the collector. In this way, we were able to measure the Lag distance, defined as the distance between the point of interest and the projection of the middle point of the nozzle tip outlet to the collector. All detected objects are denoted graphically in Fig. 3d, which shows the video output after Task 4 during the computer vision workflow.

As a next step, we asked how we could leverage the extracted features to learn the dynamics of the process in the most efficient data-driven way, with respect to both experimental and computational cost. To address this question, we developed several Bayesian learning techniques, hereafter denoted as the Machine Learning module of the GPJet framework. The Machine Learning module takes as input the extracted high-fidelity data and initially uses Gaussian Processes (GPs) to approximate the function describing the relationship between (a) the jet radius profile and the nozzle tip to collector distance and (b) the Lag distance and the ratio of the collector speed over the jet speed at the point of interest.

Gaussian process regression (GPR) is a robust statistical, non- parametric technique for function approximation with kernel machines. GPR provides the important advantages of uncertainty quantification, the ability to perform well with small datasets and the capability to easily include domain-aware physics-based models in the deployed kernels.

**Fig. 5:**
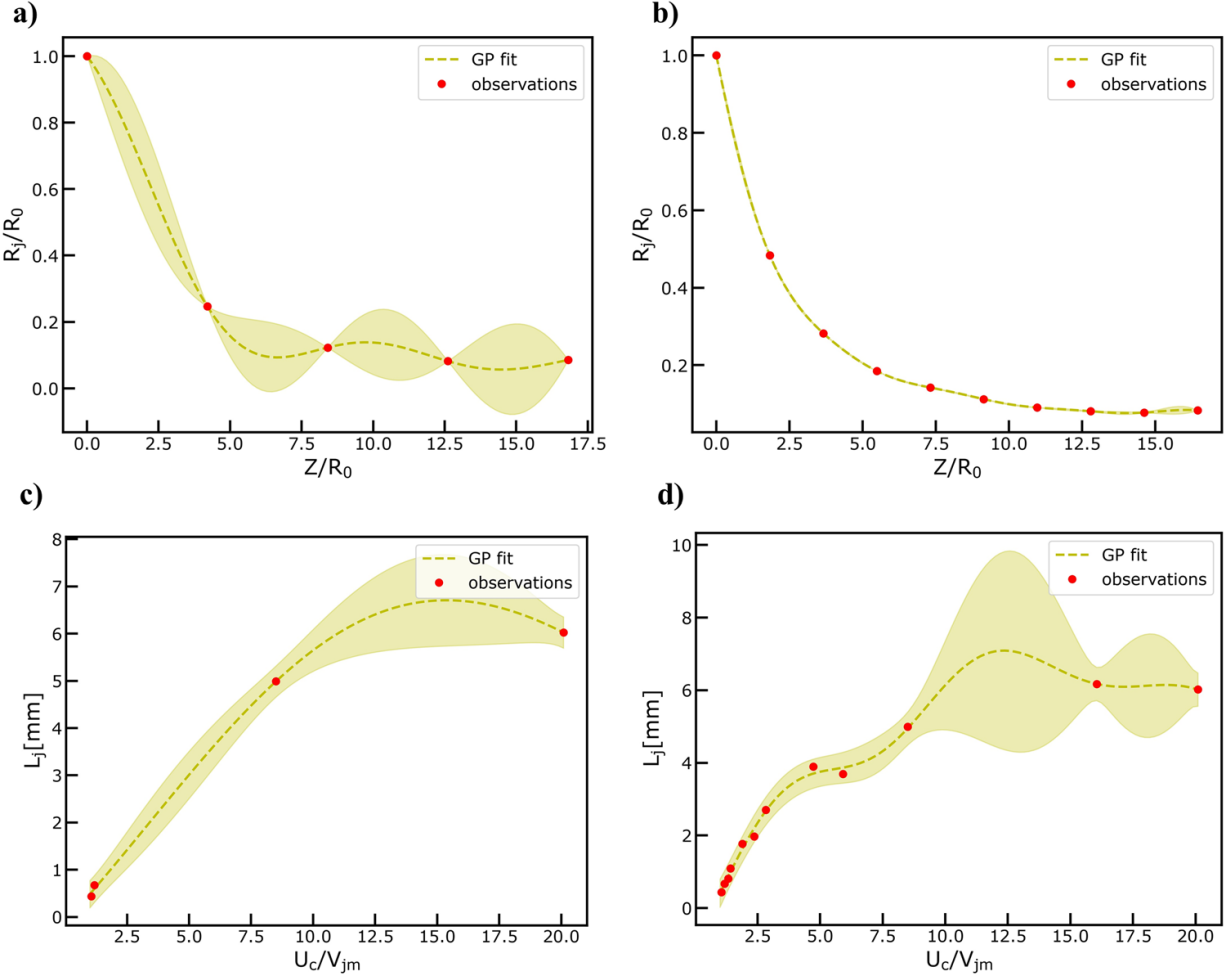
Results of Gaussian Process Modeling Regression Tasks. **a** Fitting normalized (${R}_{j}/{R}_{o}\,$) jet radius observation data (*n* = 5) obtained from the computer vision metrology module of the GPJet framework at specific z axis coordinates along the normalized jet length ($Z/{R}_{o}\,$). **b** Fitting normalized jet radius using a higher number of observation data (*n* = 10) compared to the previous case **a**. **c** Fitting lag distance (${L}_{j}$) observation data (*n* = 3) obtained from the computer vision metrology module of the GPJet framework for specific speed ratios (${U}_{c}/{V}_{{jm}}$). **d** Fitting lag distance using all available observation data (*n* = 12). For non-normalized quantities units are in SI. Filled contours (shading) represent uncertainty bounds (95% confidence intervals (CIs)).

To learn how the jet radius profile evolves over the tip to collector distance, we chose radial basis functions (RBF) as the kernel approximator and performed GPR. We trained the model under two different scenarios with *n* = 5 observations and *n* = 10 observations chosen at equally spaced points along the jet length for the 1^st^ and 2^nd^ scenario, respectively. It is important to mention that the machine vision module provides *n* = 93 observations along the jet length. The results are shown in Fig. 5a, b for the two different training scenarios. GPs can approximate the jet radius profile evolution with just *n* = 10 observations showcasing the efficiency of our data-driven approach with respect to computational cost.

To learn the function describing the relationship between the Lag distance and the ratio of the collector speed over the jet speed at the point of interest, we employ the same modeling strategy as before. Similarly, we set up two different training scenarios with *n* = 4 observations and *n* = 12 observations, respectively. Please note here that the number of high-fidelity observations at our disposal is constrained by our previously published experimental dataset (see Machine vision module under the Methods section), where videos were acquired only at 12 different speed ratio settings. The results are shown in Fig. 5c, d for the two different training scenarios. While in the 1^st^ training scenario, GPR provides a smooth function approximation, the prediction’s error from the experimental ground truth quantified by the Root Mean Square Error (RMSE), is significantly higher compared to the 2^nd^ training scenario (see Fig. S3b–d in Supplementary Information). As a result, the function describing the relationship under question is hard to approximate due to the limited available dataset that we used to test our framework. Specifically, the dataset is non-uniform across the space of the tested independent process parameters (ratio of the collector speed over the jet speed) leaving us with no data at certain regions of the space (see Fig. 5d).

Collectively, our machine vision module informing the GPR capabilities of the machine learning module with high-fidelity observations demonstrates that we can learn the dynamics of the process. Specifically, GPJet demonstrates excellent performance with respect to the prediction of jet radius profile evolution for a small amount of high-fidelity observations *n* = 10. Furthermore, GPJet demonstrates very good performance for the available number of high-fidelity observations with respect to the Lag distance behavior at different collector speed settings.

### Learning jet dynamics from videos & physics

As a next step, we focused on exploring how we could further reduce the number of high-fidelity observations without losing the predictive capability of GPR with respect to the jet radius profile evolution. To accomplish that, we augmented the high-fidelity observations obtained by the machine vision module with low-fidelity observations obtained in a principled manner by a multi-physics model. The multi-physics model captures the electro-hydrodynamics, the heat transfer and viscoelastic constitutive material behavior of the molten jet in 1D across the needle tip to collector distance. The mathematical formulation and numeric implementation of the model are described in detail in sub-section Machine learning module under the Methods sections.

We set up our data-driven scheme with two fidelities corresponding to two different kernel machines integrated in one multi-fidelity kernel, in which the correlation between the two kernels is encoded as a linear relationship. In other words, we constrain the prior knowledge during GPR with physics-relevant knowledge, resulting to a physics-informed posterior prediction that requires much less high-fidelity observations.

**Fig. 6:**
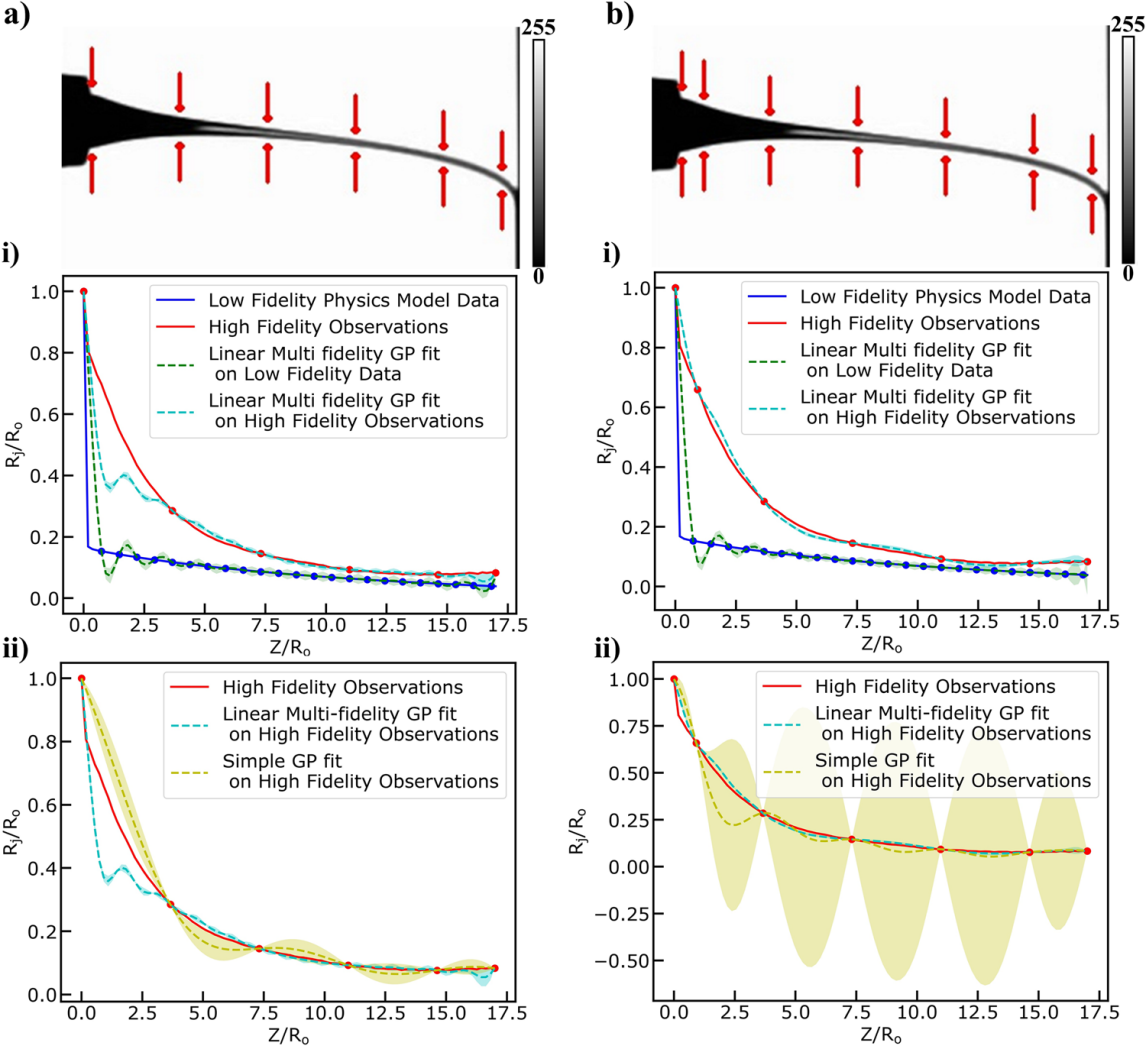
Results of Multi-fidelity Modeling Regression Tasks. **a** fitting normalized high fidelity observation data (*n* = 6, red color) of jet radius (${R}_{j}/{R}_{o}\,$) and low fidelity model data obtained from the computer vision metrology module of the GPJet framework and from the multi-physics model, respectively, at specific z axis coordinates along the normalized jet length ($Z/{R}_{o}\,$) and comparing the results with a simple GP fit using the same number of high fidelity observation data. **b** fitting a higher number of normalized high fidelity observation data (*n* = 7, red color) of jet radius (${R}_{j}/{R}_{o}\,$) and low fidelity model data obtained from the computer vision metrology module of the GPJet framework and from the multi-physics model, respectively, at specific z axis coordinates along the normalized jet length ($Z/{R}_{o}\,$) and comparing the results with a simple GP fit using the same number of high fidelity observation data. Filled contours (shading) represent uncertainty bounds (95% confidence intervals (CIs)). The jet profile images in **a**, **b** are images in grayscale (0–255) with the 0 value and the 255 value in the color bar representing the black jet profile, and the white background, respectively.

We trained the multi-fidelity model under two different scenarios with *n* = 6 high-fidelity observations and *n* = 7 high-fidelity observations, respectively. For both scenarios the number of low-fidelity observations was kept to a number equal to 32 and equally spaced points across the jet length. For the 1st scenario *n* = 6 equally spaced points were chosen across the jet length depicted in the jet schematic of Fig. 6a (upper left). The results are shown in Fig. 6ai and Fig. 6aii. In Fig. 6ai, we plot the multi-fidelity GPR predictions for the low and high-fidelity observations respectively. In Fig. 6aii, we plot the predictions of the multi-fidelity GPR in high-fidelity observations together with the predictions of a simple GP in high-fidelity observations. Both plots demonstrate that we can learn the jet radius profile much better using two different fidelities compared to using only one fidelity for the same number of high-fidelity observations. Our results, point out that we lose predictive accuracy for the Taylor cone area (below the needle tip outlet). This phenomenon was expected due to that the fact that similar behavior was observed when the multi-physics model was tested and informed the strategy of the 2^nd^ scenario, where we chose 7 high-fidelity observations with the additional point being in the Taylor cone area. The results are shown in Fig. 6bi and Fig. 6bii demonstrating that we have managed to further reduce the required number of high-fidelity observations that need to be extracted by the machine vision module without compromising the predictive accuracy.

### Active learning of jet dynamics

Up to now, we demonstrated that GPJet, is a robust tool for passive learning of jet dynamics. By “passive”, we mean that given a high-fidelity dataset provided by the Machine Vision module and augmented by low-fidelity data provided by the Physics-based module, the GPR capabilities of the Machine Learning module can model the function that mathematically represents the relation between the jet radius and the needle tip to collector distance. In addition to that, we employed the same strategy without low fidelity data, to model the function describing the highly dynamic relationship between the Lag distance and the ratio of the collector speed and the jet velocity at the point of interest.

In this section, we asked the questions of whether we could actively choose data points across jet length for which to observe the outputs to accurately model the underlying function describing the jet dynamics with respect to the extracted jet features. To accomplish that, we deploy a virtual MEW machine, whose dynamic range is defined by the available dataset, and we run simulation experiments to demonstrate if we can learn the underlying functions in an active manner as quickly and accurately as possible.

**Fig. 7:**
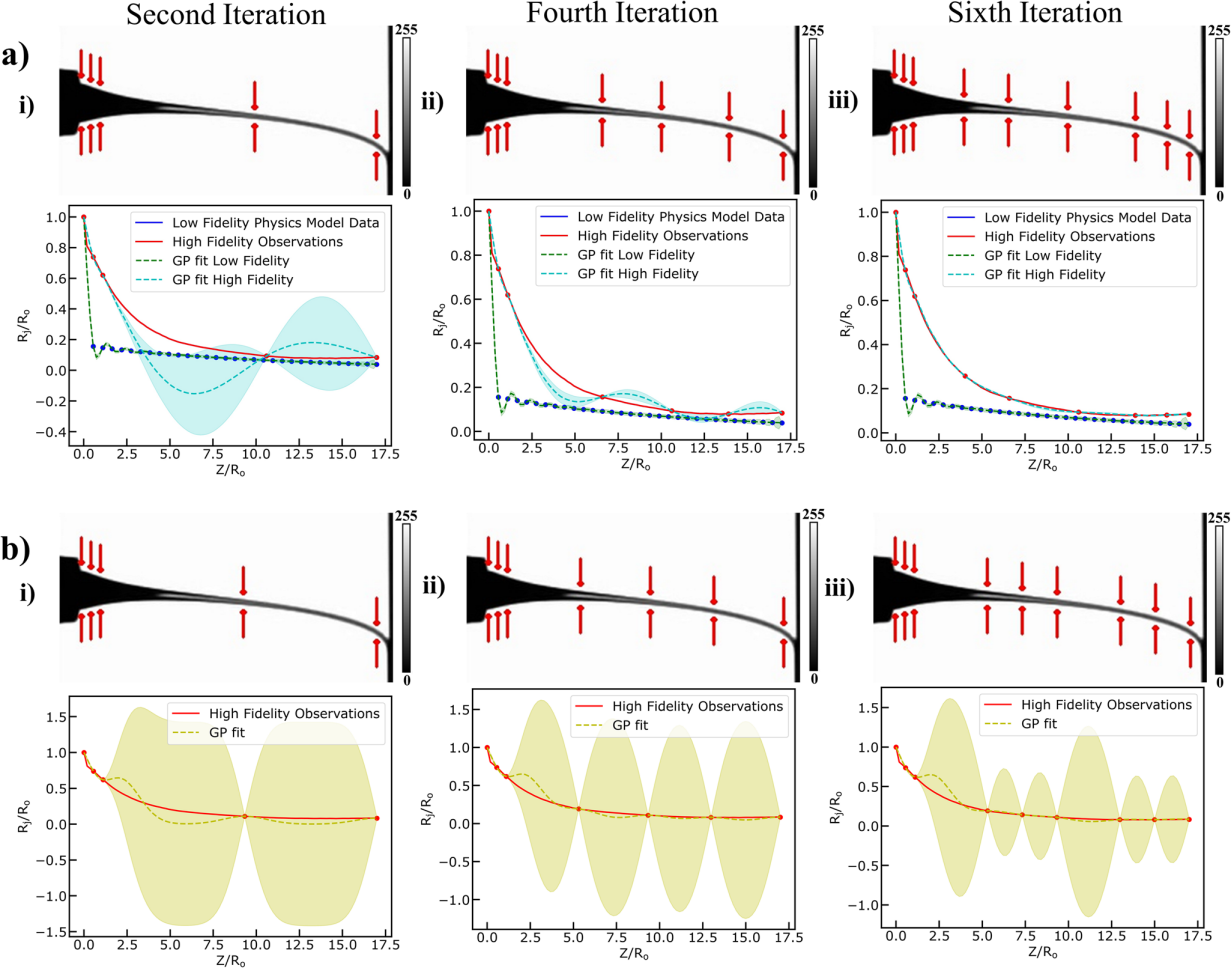
Results of Active Learning Task on Multifidelity Data versus on only High-Fidelity Data. **a** Exploring the design space using Active Learning to fit a Multifidelity Gaussian Process to normalized high fidelity observation data (red color) of jet radius (${R}_{j}/{R}_{o}\,$) and low fidelity model data obtained from the computer vision metrology module of the GPJet framework and from the multi-physics model, respectively, at specific z axis coordinates along the normalized jet length ($Z/{R}_{o}\,$), (**i–iii**) denote the second, fourth and sixth (final) iteration of the active learning algorithm until it meets its termination criteria. The points across the jet assessed at each iteration are pointed with red arrows. **b** exploring the design space using Active Learning to fit a Gaussian Process to normalized high fidelity observation data (red color) of jet radius (${R}_{j}/{R}_{o}\,$) obtained from the computer vision metrology module of the GPJet framework at specific z axis coordinates along the normalized jet length ($Z/{R}_{o}\,$), (**i–iii**) denote the second, fourth and sixth (final) iteration of the active learning algorithm. The points across the jet assessed at each iteration are pointed with red arrows. Filled contours (shading) represent uncertainty bounds (95% confidence intervals (CIs)). The jet profile images in **a**, **b** are images in grayscale (0–255) with the 0 value and the 255 value in the color bar representing the black jet profile, and the white background, respectively.

To accomplish that, we set up an exploration scenario, a set-up closely related to optimal experimental design scenarios as it equates to adaptively selecting the input spatial points across the jet length based on what is already known about the function describing the jet radius profile and where knowledge can be improved. We run active learning in both the multi-fidelity GP and simple GP for the jet radius profile evolution. The results are shown in Fig. 7. To systematically, compare the performance of the two different models, we chose the same initial training points (Fig. S5a-i and S5b-i) and the same number of iterations during each training phase. For each iteration (Fig. S5a(i-vi) and S5b(i–vi)), we graphically show, on the processed video frame the adaptively selected point across the jet length and below that the modeling results. The adaptive selection is based on a purely exploratory acquisition function that steers the point selection towards the area of least knowledge quantified by the uncertainty output of the modeling step. The results demonstrate that we can learn actively and in a purely exploratory scenario accurately and fast the underlying function. Each iteration phase for the multi-fidelity (MFD) GPs and simple GPs lasts around ~0.5 s leading to a total learning time equal to 3 s. Lastly, we extract performance metrics to compare the active learning between the multi-fidelity and simple GP model (see Fig. S2a–c in Supplementary Information). The results demonstrate that active learning on the MFD model is significantly faster (Fig. 2a–c) with more confident predictions since the model’s prior assumptions are constrained by domain-aware data.

**Fig. 8:**
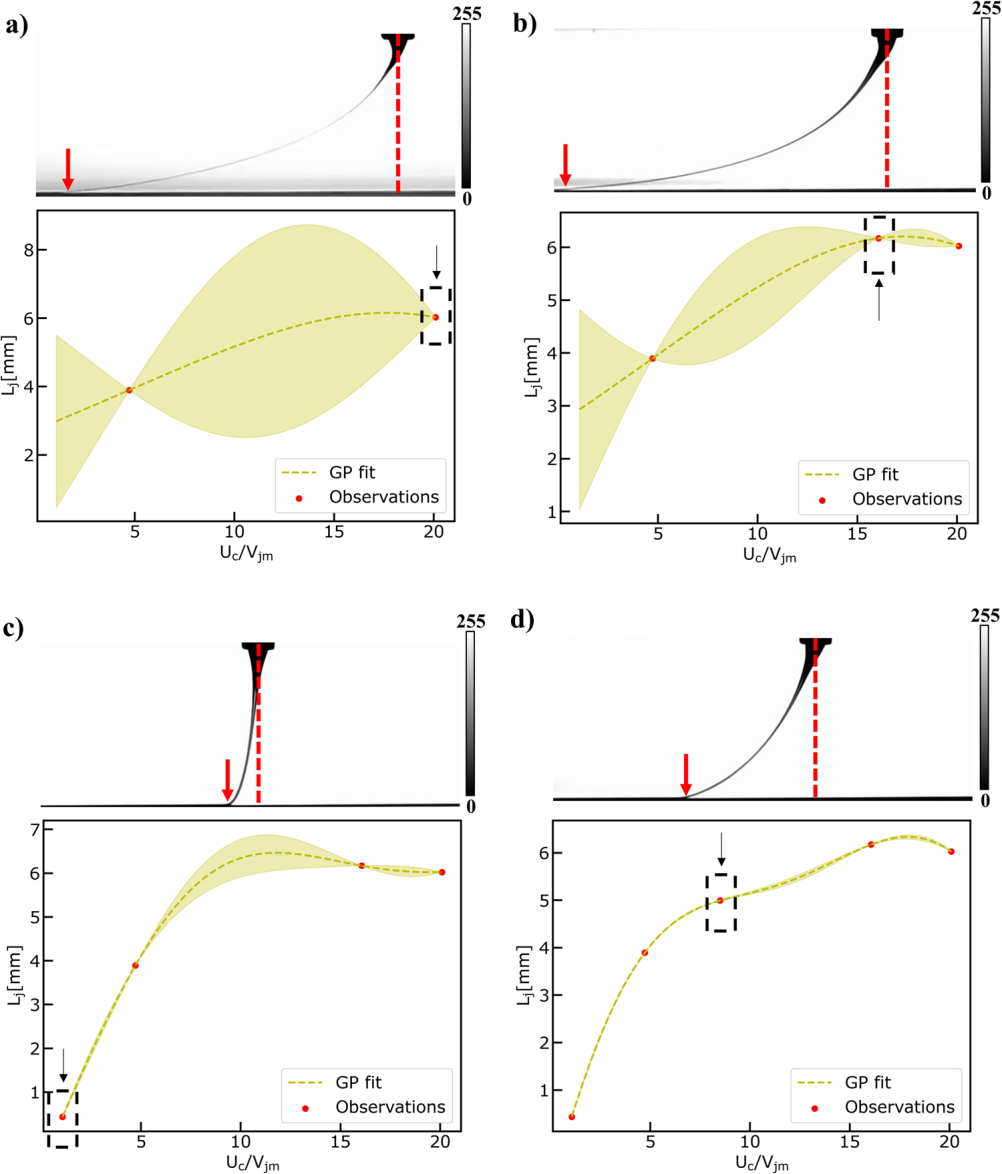
Results of Exploring the Design Space Task. Exploring the design space using active learning to fit a Gaussian Process Model to lag distance (${L}_{j}$) observation data obtained from the computer vision metrology module of the GPJet framework (the distance between the red arrow and the red dashed line) for specific speed ratios (${U}_{c}/{V}_{{jm}}$). **a**–**d** Iterations of the active learning algorithm until it meets termination criteria. In every case, the observation point chosen at each iteration is denoted with a black dashed line box pointed by a black arrow. Filled contours (shading) represent uncertainty bounds (95% confidence intervals (CIs)). The jet profile images in a), b), c) and d) are images in grayscale (0–255) with the 0 value and the 255 value in the color bar representing the black jet profile, and the white background, respectively.

Then, we employ the same strategy to actively learn the function describing the relation between the Lag distance and the speed ratio (put symbol) in an exploration scenario. The results are shown in Fig. 8. The virtual MEW machine performs remarkably well in the prescribed experimental simulation. It starts by randomly selecting one speed ratio equal to 5 (see Fig. 8a) and after 4 additional iterations (see Fig. 8a–d), the underlying function is quite effectively approximated. Performance metrics (see Fig. S3b–d and Fig. S4 in Supplementary Information) demonstrate that the underlying function can be learned fast in an active manner and provide predictions with higher confidence compared to the passive learning approach and specifically after training the GP with all the available high-fidelity observations.

**Fig. 9:**
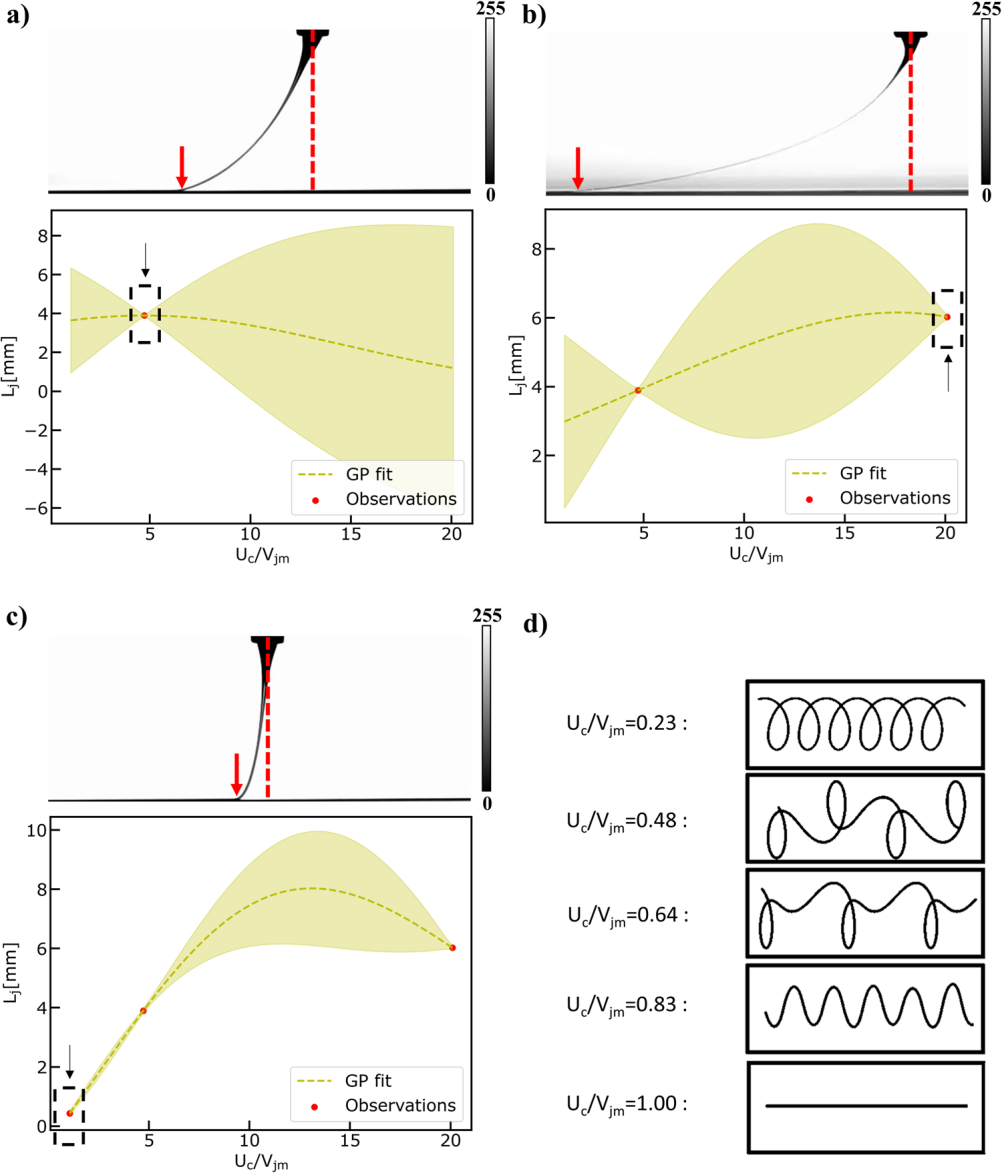
Results of Bayesian Optimization Task. Performing Bayesian Optimization to find the minimum lag-distance (${L}_{j}$) by fitting a Gaussian Process Model to lag distance (${L}_{j}$) observation data obtained from the computer vision metrology module of the GPJet framework (the distance between the red arrow and the red dashed line) for specific speed ratios (${U}_{c}/{V}_{{jm}}$). **a**–**c** Iterations of the Bayesian optimization algorithm until it meets termination criteria. In every case, the observation point chosen at each iteration is denoted with a black dashed line box pointed by a black arrow. **d** For speed ratios less than one $({U}_{c}/{V}_{{jm}} < 1)$ the process is unstable, no straight line is formed, instead the translated coiling, alternating loops, W patterns and meanders patterns are formed (depicted with a black line), for ${U}_{c}/{V}_{{jm}}=0.23,{U}_{c}/{V}_{{jm}}=0.48,\,{U}_{c}/{V}_{{jm}}=0.64,{U}_{c}/{V}_{{jm}}=0.83$ respectively. Therefore, no lag distance (${L}_{j}$) observation data can be obtained from the computer vision metrology module of the GPJet framework. Filled contours (shading) represent uncertainty bounds (95% confidence intervals (CIs)). The jet profile images in **a**, **b** and **c** are images in grayscale (0–255) with the 0 value and the 255 value in the color bar representing the black jet profile, and the white background, respectively.

Finally, we set out to address the following question. Can the virtual MEW machine find the speed ratio corresponding to the minimum Lag distance in an autonomous way? Autonomy in this paper, refers to the machine’s ability to self-drive measurements of an experiment. Some initial parameters, such as the parameters to explore and their corresponding ranges constrained by the dataset, is defined by the user a priori. Instead of us learning the relation between the Lag distance and the speed ratio and afterwards calibrating the machine hyperparameters, we aim to demonstrate a self-calibrating scenario. To achieve that we employ an exploitation-exploration strategy in the spirit of Bayesian Optimization (BO). It is called exploration–exploitation as scenarios where the output of the underlying function must be optimized require us to both sample uncertain areas to acquire more knowledge about the function (exploration) as well as sampling input points that are likely to produce extremum outputs given the current knowledge of the function (exploitation). The virtual MEW machine performs remarkably well in the prescribed experimental simulation. It starts again by randomly selecting a speed ratio equal to (see Fig. 9a) and after 2 additional iterations (see Fig. 9a–c) the speed ratio corresponding to the minimum Lag distance has been reached. This speed ratio is close to 1, as expected from the mechanical sewing machine model, which is described in detail in Physics-based modeling module under the Methods section. BO validates the initial hypothesis formed by universality about the mechanical sewing machine model.

## Conclusions

In this work, we demonstrate GPJet, an end-to-end physics-informed probabilistic machine learning framework that sets the basis for the next generation of self-calibrating E-jet printing machines. We construct a virtual melt electrowriting (MEW) machine using a previously published video dataset acquired by a conventional camera that performed online jet monitoring under various process conditions. We demonstrate that GPJet can extract high-fidelity jet features in near real time from the video data using a highly efficient computer vision algorithmic workflow that is implemented in a hybrid multiprocessing—multithreading approach. Additionally, two physics-based models were implemented, providing efficiently prior process physics knowledge, in the form of low-fidelity data. The first one can predict the evolution of the jet across the free-flow regime while the second one can predict the deposition dynamics of a gravity-driven viscous thread onto a slowly moving surface known as the “fluid-mechanical sewing machine”. Furthermore, we set out to learn process dynamics with minimum experimental cost, as described by the required number of high-fidelity data. To accomplish that, a probabilistic machine learning module was developed based on Gaussian process regression (GPR) as the surrogate modeling step, active learning for pure process dynamics exploration and Bayesian optimization for process optimization. Two case studies were performed, one regarding the jet diameter profile and the other one regarding the lag distance. Our results demonstrate that for an offline learning strategy, the number of data and their respective position in the design space are crucial for the quality and the confidence of the predictions in both cases. Also, in the case of jet radius profile, a multi-fidelity GPR modeling approach coupling high-fidelity data from the machine vision module, with low-fidelity data from the physics-based jet evolution model, can provide better and more confident predictions, while using less high-fidelity observations. Incorporating prior physics knowledge leads to computational cost reduction, since jet diameter needs to be evaluated in less points across the nozzle to bed distance, and thus to even faster video processing times during the high-fidelity feature extraction step. As a next step, an online learning strategy was employed to actively learn the jet diameter profile with and without multi-fidelity modeling. Importantly, we demonstrate that we can effectively learn jet evolution more accurately in the online learning compared to the offline learning scenario when it is informed by physics guided by the variance. Finally, in an online calibration scenario, the Optimizer managed to minimize the lag distance, by fine-tuning the collector’s speed.

GPJet serves as an important step towards autonomous self-calibrating E-jet printing processes by integrating machine learning models that offer (a) uncertainty quantification for decision making after the modeling step and (b) lower fidelity physics-based models for higher computational efficiency during online deployment. It is important to recognize the current limitations of GPJet and the challenges that we are trying to overcome with our ongoing work. In this study, we are bounded by the previously published video dataset that we used to test our framework. We are building our own physical automated manufacturing system. This will allow us to perform self-calibration experiments by setting the machine to be guided by GPJet and actively search for jet stability conditions with prescribed fiber diameter values over the whole dynamic range of each independent process parameter. Furthermore, the updating of the Machine Vision module with more robust algorithms in the future is imperative for generalized use by the whole family of E-jet printing technologies. For example, the adoption of additional functions will be adopted to allow detection of the transition from the nozzle diameter to the jet diameter, providing differentiation of the two features and tracking of the jet as it moves toward/away from the collector. We plan to include these updates in GPJet to explore its robustness beyond steady state printing conditions including the transient behavior of the jet during the initial jet formation, where we expect unseen jet instability phenomena, such as fiber breakage and beads across the jet length. GPJet can be easily integrated and guide any physical E-jet printing machine using a bi-directional network communication protocol. The jet features extracted by the Machine Vision module between each experimental run, are fed to the Machine Learning module that gives as an output a set of instructions containing the values of the recommended independent process parameters that are then fed to the machine’s control platform through the serial port. Lastly, the generalization of Gaussian processes beyond their training data given the uncertainty property rests entirely on the choice of kernel that shapes our prior belief. Incorporating prior-physics knowledge allowed us to choose radial basis functions, whose exponential nature correlated well with our physics-based model. Despite the limits of the available dataset, we have demonstrated the utility of GPJet as an automated online calibration tool that is powered by process-relevant data of multiple fidelities presenting a large step toward the autonomy of e-jet printing.

## Methods

### Machine vision module

#### Jet metrology

For the implementation of the Jet Metrology algorithm, Python 3.8 was used, along with the python bindings of the OpenCV library, which enables us to read and process video data. The jet metrology algorithm consists of two sub-algorithms. The first is the object segmentation and detection algorithm. The second is the feature extraction algorithm.

The first sub-algorithm segments the needle tip, the Taylor cone, the jet and the deposited fiber ‘on the collector. In addition to that, the algorithm attempts to find the jet’s deposition point on the collector. Finally, the segmented objects of interest are plotted for the user to visually inspect the output and assess the performance of the algorithm. To detect the objects of interest in each video frame we use the very much alike *meanshift*^31^ and *camshift*^32^ algorithms.

The *meanshift* algorithm is based on a statistical concept directly related to clustering. Similar to other clustering algorithms, the *meanshift* algorithm scans the whole frame for high concentration of pixels of the same color. The main difference between the *meanshift* and the *camshift* algorithms is that the *camshift* algorithm has the capability to adjust, so that the tracking box can change its size and direction, to better correlate with the movements of the tracked object. The *meanshift* and *camshift* algorithm are useful tools to employ for object tracking. Also, unlike neural networks and other machine learning methods for object detection, these algorithms can be immediately implemented and deployed unsupervised, i.e., without the need to train a model with numerous labeled images. Instead, the algorithm takes as an input the initial color of the object, that needs to be detected, and then it tracks it throughout the rest of the video. On the other hand, using color as a primary method of identification, neither of the two algorithms can identify objects based on specific shapes and features, which makes them less powerful than other methods. Furthermore, objects varying in color on a large scale and complex or noisy backgrounds can make object detection and tracking problematic. As a result, the *meanshift* and *camshift* algorithms work best under controlled environments.

The first step is to reverse the image colors so that the objects of interest are white and the background black. The next step is to apply a multi-color mask to segment them, and then to change the image color-space from Blue, Green, Red (BGR) to Hue, Saturation, Value (HSV). Finally, the *meanshift* algorithm is applied to detect the needle and the Taylor cone and the *camshift* algorithm to detect and track the jet.

To find the deposition point, the algorithm needs to know the collector’s position. Then, it creates a window around the collector, crops the region of interest from the frame and processes that instead of the whole frame. The built-in function used to find the deposition point is the *cv2.goodFeaturesToTrack*. This function finds the most prominent corner in our region of interest by calculating its eigen-values, as described in^33^.

Finally, by subtracting the deposition point from the nozzle’s position (center of blue rectangle in Fig. 3c), we get the lag distance, which is depicted with a two-way orange arrow in Fig. 3c.

The second sub-algorithm is the one responsible for extracting all the jet features that are relevant to the process dynamics. These features are the diameter, areas, and angles of the jet as we move along the z-axis. Another important feature is the velocity of each jet’s point along the x-axis relatively to the nozzle’s position. To get all those features we follow a straightforward procedure. The algorithm takes three inputs, the first is the current video frame. The second input is the calibration factor (${cf}$), which is a correlation between distance units (mm) and pixels. The last one is the stride. The stride indicates every how many pixels along the z-axis we perform computations. Using too small a stride would lead to more precise calculations but would tremendously increase the computation time. On the other hand, using too large a stride would lead to shorter computation times but at a risk to lose important information.

The first step is to change the frame’s color-space from RGB scale to grayscale, so that the Canny edge detection algorithm^34^ can be applied. The parameters of the Canny edge detector are [threshold_1, threshold_2] and were specified to 150 and 255 in a semi-automatic way, using trackbars while performing edge detection to other video samples. After performing Canny edge detection, we read the first row of pixels in our canny frame, which now is an array of 0 and 255. If Canny algorithm has been implemented correctly when we read this row of pixels from left to right, the first time we encounter a 255 should be the left edge (${le}$) of our jet. Likewise, the first time we encounter a 255 while reading the row of pixels from right to the left, should be the right edge (${re}$) of our jet. By subtracting those two pixels’ indices and multiplying with the calibration factor we get the diameter of the jet at this position in the z-axis, which is equal to $2\,{R}_{j}$:

1 $${{{{{\rm{Jet}}}}}}\; {{{{{\rm{Radius}}}}}}\;\;\;\;\;\;\;\;\;\;2{R}_{j}=\left({re}-{le}\right)* {cf}$$

Those indexes are also stored in two variables ($l{e}_{{previous}},{r}{e}_{{previous}}$) so that they can be used to calculate the jet angles as we move down the z-axis. Then we repeat the procedure for every ‘stride’ rows. After finding the left (${le}$) and right (${re}$) edges and calculating the diameter, the area and angles can be calculated as:

2 $${{{{{\rm{Jet}}}}}}\; {{{{{\rm{Area}}}}}}\;\;\;\;\;\;A_{j}\,{=[(({re}_{previous}-{le}_{previous})-({re}-{le}))* {stride}]* {cf}}^{2}$$

3 $${{{{{\rm{Left}}}}}}\; {{{{{\rm{Jet}}}}}}\; {{{{{\rm{Angle}}}}}}\;\;\;\;\;\;\;\;\;\;\;\;\;\;\;\;\;\;\;\;\;\;\;\;\;\;\;\;{\theta }_{{jl}}=\arctan (({le}-{le}_{previous})/{stride})$$

4 $${{{{{\rm{Right}}}}}}\; {{{{{\rm{Jet}}}}}}\; {{{{{\rm{Angle}}}}}}\;\;\;\;\;\;\;\;\;\;\;\;\;\;\;\;\;\;\;\;\;\;\;\;{\theta }_{{jr}}=\arctan (({re}\,-{re}_{previous})/{stride})$$

5 $${{{{{\rm{Jet}}}}}}\,{{{{{\rm{Velocity}}}}}}\;\;\;\;\;\;\;\;\;\;\;\;\;{U}_{j}=({{re}}_{{current}{frame}}-{{re}}_{{previous}{frame}})* ({cf}/(1/{fps}))$$

The $l{e}_{{previous}},{r}{e}_{{previous}}$ are then updated with the ${le},{re}$ values. After accessing all frame’s rows, the algorithm returns arrays containing all the quantified diameters, areas, right boundaries, angles left and angles right. The same procedure is applied to all frames. Right boundaries are important because by subtracting the right edges of two consecutive frames we can calculate the jet’s velocity $({U}_{j})$ on the x-axis.

### Physics-based modeling module

#### Multiphysics model

The importance of accurately extracting jet properties is signified by several studies on predicting the jet stable region diameter, through mathematical modeling. Zhmayev et al. proposed a model by fully coupling the conservation of mass, momentum, charge and energy equations with a constitutive model and the electric field equations at the steady state^33^. Similar to most models, they utilize the thin filament approximation to obtain a simpler and more tractable solution. This assumption is possible by appropriately averaging the model variables across the radial direction. In addition, the charge and electric field equations are simplified, under the assumption of low electrical conductivity, as compared to the governing equations for isothermal simulations presented by Carroll and Joo^34^. The conservation of energy relation and a non-isothermal constitutive model were added to extend to non-isothermal situations. The resulting governing equations after being nondimensionalized are as follows (see Table S5 in Supplementary Information):

6 $${{{{{\bf{Continuity}}}}}}{{{{{\boldsymbol{:}}}}}}\,{R}_{j}^{2}{V}_{j}=1$$

7 $${{{{{\bf{Momentum}}}}}}{{{{{\boldsymbol{:}}}}}}\, {Re}{V}_{j}{V}_{j}^{{\prime} }= {Bo}	+\frac{{\left({R}_{j}^{2}\left({\tau }_{{zz}}-{\tau }_{{rr}}\right)\right)}^{{\prime} }}{{R}_{j}^{2}}+\frac{{R}_{j}^{{\prime} }}{{Ca}{R}_{j}^{2}}\\ 	+{F}_{e}\left[\sigma {\sigma }^{{\prime} }+{\beta }_{{\rm E}}{{\rm E}}_{t}{E}_{t}^{{\prime} }+\frac{2\sigma {{\rm E}}_{t}}{{R}_{j}}\right]$$

8 $${{{{{\bf{Charge}}}}}}{{{{{\boldsymbol{:}}}}}}\,\sigma =R$$

$${{{{{\bf{Electric\; field}}}}}}{{{{{\boldsymbol{:}}}}}}\,{E}_{t}=\frac{1}{\left(1+2Z-\frac{{Z}^{2}}{x}\right)\left(\sqrt{1+{\left({R}_{j}^{{\prime} }\right)}^{2}}\right)}$$

9 $$\hskip 55pt {E{\prime} }_{t}=\frac{-2+2Z/x}{\left(1+2Z-\frac{{Z}^{2}}{x}\right)\left(\sqrt{1+{\left({R}_{j}^{{\prime} }\right)}^{2}}\right)}$$

10 $${{{{{\bf{Energy}}}}}}{{{{{\boldsymbol{:}}}}}}\,{Pe}{V}_{j}{\varTheta }^{{\prime} }={Na}{V}_{j}^{{\prime} }\left({\tau }_{{zz}}-{\tau }_{{rr}}\right)-\frac{2B{i}_{L}\left(\varTheta -{\varTheta }_{{{{{{\rm{\infty }}}}}}}\right)}{{R}_{j}}$$

11 $${{{{{\bf{Constitutive}}}}}}{{{{{\boldsymbol{:}}}}}}\,{\tau }_{{zz}} = \, 	 {\tau }_{p,{zz}}+2\beta f\left(\varTheta \right){V}_{j}^{\prime} \,{\tau }_{{rr}}={\tau }_{p,{rr}}-\beta f\left(\varTheta \right){V}_{j}^{{\prime} }\,{\tau }_{p,{zz}}\\ 	 + \,\frac{{De}\varGamma }{\Theta +\varGamma }\left(\frac{\alpha {\tau }_{p,{zz}}^{2}}{1-\beta }+f\left(\varTheta \right)\left[{V}_{j}{\tau }_{p,{zz}}^{{\prime} }-2{V}_{j}^{{\prime} }{\tau }_{p,{zz}}-\frac{{V}_{j}{\tau }_{p,{zz}}{\varTheta }^{{\prime} }}{\varTheta +\varGamma }\right]\right)\\ = \,	2\left(1-\beta \right)f\left(\varTheta \right){V}_{j}^{{\prime} }\,{\tau }_{p,{rr}}+\frac{{De}\varGamma }{\varTheta +\varGamma }\left(\frac{\alpha {\tau }_{p,{rr}}^{2}}{1-\beta }+f\left(\varTheta \right)\left[{V}_{j}{\tau }_{p,{rr}}^{{\prime} }+{V}_{j}^{{\prime} }{\tau }_{p,{rr}}\right]\right)\\ = \,	2\left(1-\beta \right)f\left(\varTheta \right){V}_{j}^{{\prime} }f\left(\varTheta \right)=\exp \exp \left[\frac{\varDelta {\rm H}}{{R}_{{ig}}\varDelta {{ T}}_{{Rh}}}\left(\frac{1}{\varTheta +\varGamma }-\frac{1}{\varGamma }\right)\right]$$

The system of equations can be reduced to a set of five coupled first order ordinary differential equations (ODEs). Boundary Conditions are required, in order to proceed towards the numerical solution.

12 $${\tau }_{p,{zz}}{{{{{{\rm{|}}}}}}}_{Z=0}=2\left(1-\beta \right)f\left(\varTheta \right){V}_{j}^{\prime}$$

13 $${\tau }_{p,{rr}}{{{{{{\rm{|}}}}}}}_{Z=0}=-\left(1-\beta \right)f\left(\varTheta \right){V}_{j}^{\prime}$$

14 $$\varTheta {|}_{Z=0}=0$$

15 $$R{|}_{Z=0}=1$$

16 $${\left[\frac{6}{{R}_{j}^{4}}{\left({R}_{j}^{{\prime} }\right)}^{2}+\left(\frac{1}{{Ca}{R}_{j}^{2}}+{Fe}{R}_{j}\right){R}_{j}^{{\prime} }+\frac{2{Fe}}{\sqrt{1+{\left({R}_{j}^{{\prime} }\right)}^{2}}}\left(1-\frac{{\beta }_{{ E}}}{\sqrt{1+{\left({R}_{j}^{{\prime} }\right)}^{2}}}\right)\right]}_{z=0}=0$$

The model was implemented in Python. While true properties and parameters of the material are not provided the ones used in ref. ^13^ for PCL were used and are presented in Tables S2 and S3. As also referred in refs. ^12,13^^,^ the model slightly underpredicts the jet radius while in the Taylor cone area, but when the jet is stabilized, it accurately predicts it’s radius. Knowing this, even if the volumetric flowrate ($Q$) is not provided with the dataset, a Particle Swarm Optimization (PSO) algorithm was also implemented to find the $Q$ for which the predicted jet’s radius better fits the computer vision observations.

#### Geometrical model

Lag distance is a highly important parameter regarding the quality of the process outcome. Specifically, for some collector speeds, the jet falls onto the moving collector in a way reminiscent of a sewing machine, generating a rich variety of periodic patterns, such as meanders, W patterns, alternating loops and translated coiling (see Fig. 9d). Brun et al.^35^ proposed a quasistatic geometrical model, consisting of three coupled ordinary differential equations for the radial deflection, the orientation, and the curvature of the path of the jet’s contact point with the collector, capable of reconstructing the patterns observed experimentally while successfully calculated the bifurcation threshold of different patterns. They also evidenced that the jet/collector velocity ratio (${U}_{c}/{V}_{{jm}}$) was the key factor for pattern variation.

According to this geometrical model, the deposited trace on the collector is a combination of the obit of the contact point (when collector’s speed is equal to zero ${U}_{c}=0,$ the jet creates coiling patters with radius ${R}_{c}$) and the movement of the collector.

17 $$q\left(s,t\right)=r\left(s\right)+{U}_{c}\left(t-\frac{s}{{V}_{{jm}}}\right){e}_{x},$$

where $q(s,t)$ is the deposited trace, *s* is the arc-length, *t* is time, $r(s)$ is the contact point at time *s*/*V*_*jm*_, *e*_*x*_ is the direction of the collector’s speed, $t-s/{{V}_{jm}}$ is the time that the contact point moves together with the collector. Differentiating $q\left(s,t\right)$ and moving from Cartesian to Polar coordinates ($r,\psi$ denote the polar coordinates of the contact point $r(s)$), and considering the curvature ${\theta }^{{\prime} }$ at the bottom of the jet, we get the system of ODEs:

18 $${r}^{{\prime} }=\cos \cos \left(\theta -\psi \right)+\frac{{U}_{c}}{{V}_{{jm}}}\cos \psi$$

19 $${\psi }^{{\prime} }=\frac{1}{r}\left(\sin \sin \left(\theta -\psi \right)-\frac{{U}_{c}}{{V}_{{jm}}}\sin \psi \right)$$

20 $${\theta }^{{\prime} }=\frac{1}{{R}_{c}}\sqrt{\frac{r}{{R}_{c}}}\left(1+\frac{{0.715}^{2}\cos \cos \left(\theta -\psi \right)}{1-0.715\cos \cos \left(\theta -\psi \right)}r\right)\sin \sin \left(\theta -\psi \right)$$

This geometrical model was implemented in Python and by varying the dimensionless parameter ${U}_{c}/{V}_{{jm}}$ from 0 to 1 as suggested^30^, the orbit and the deposited trace can be reconstructed. Verifying the results from^30^, the critical velocity at which the straight pattern appears is ${U}_{c}={V}_{{jm}}$, which means ${U}_{c}/{V}_{{jm}}=1$. for speed ratios ${0 < U}_{c}/{V}_{{jm}} < 1$ the process is highly unstable, forming the translated coiling, alternating loops, W patterns and meanders when the speed ratios are 0.23, 0.48, 0.64, 0.83, respectively.

### Machine learning module

#### Gaussian process regression

Gaussian process regression is a non-parametric stochastic process with strong probabilistic establishment^35^. GPR is a supervised machine learning technique, which predicts a probability distribution based on Bayesian theory unlike other machine learning algorithms that give deterministic predictions. The idea behind GPR is that the posterior probability can be modified based on a prior probability, given a new observation. Those characteristics allow the uncertainty quantification of each point prediction. Assuming there is a dataset available, consisting of input-output pairs of observations $D=\left\{{x}_{i},{y}_{i}\right\}=\left(x,y\right),{i}=1,\,2,\,\ldots ,{n}$ that are generated by an unknown model function $f$

21 $$y=f\left(x\right),\,x\epsilon {R}^{d}$$

$f\left(x\right)$ can be completely estimated by a mean $m\left(x\right)$ and a covariance function $K\left(x,{x{{\hbox{'}}}}\right).$

22 $$m\left(x\right)=E[\,f\left(x\right)]$$

23 $$K\left(x,x{\prime} \right)=E[\left(f\left(x\right)-m\left(x\right)\right)\left(f\left({x}^{{\prime} }\right)-m\left({x}^{{\prime} }\right)\right)]$$

GPR aims to learn the mapping between the set of input variables and the unknown model *f(x)*, given the set of observations *D*. To map this correlation *f(x)* is typically assigned a GP prior.

Gaussian processes (GPs) are powerful modeling frameworks incorporating a variety of kernels. A Gaussian Process is a collection of random variables, any finite number of which have a joint Gaussian distribution^35^.

24 $$f \sim {GP}\left(m\left(x\right),k\left(x,{x}^{{\prime} }{{{{{\rm{;}}}}}}\theta \right)\right)$$

where $k$ is a kernel function with a set of trainable hyperparameters $\theta$. The kernel defines a symmetric-positive covariance matrix ${K}_{{ij}}=k({x}_{i},{x}_{j};\theta ),\,{ K}\epsilon {R}^{{nxn}}$, which reflects the prior available knowledge on the function to be approximated. Furthermore, kernel’s eigenvalues define a reproducing kernel Hilbert space, that determines the class of functions within approximation capacity of the predictive GP posterior mean. Hyper-parameters $\theta$ are trained by maximizing the marginal log-likelihood of the model^35^.

Assuming a Gaussian likelihood and using the Sherman–Morrison–Woodbury formula the expression for the posterior distribution $p({f|y},X)$ is tractable and can be used to perform prediction given a new output ${f}_{n+1}$ for a new input ${x}_{n+1}$.

26 $$p\left({y}_{1:n},{x}_{1:n},{x}_{n+1}\right)=N\left({\mu }_{n}\left({x}_{n+1}\right),{\sigma }_{n}^{2}\left({x}_{n+1}\right)\right)$$

27 $${\mu }_{n}\left({x}_{n+1}\right)={k}_{n+1}{K}^{-1}{y}_{1:n}$$

28 $${\sigma }_{n}^{2}\left({x}_{n+1}\right)=k({x}_{n+1},{x}_{n+1})-{k}_{n+1}{K}^{-1}{k}_{n+1}^{T}$$

where ${k}_{n+1}=\left[k\left({x}_{n+1},\,{x}_{1}\right),\,\ldots ,{k}\left({x}_{n+1},{x}_{n}\right)\right]$. As referenced before prediction consists of a mean, computed using the posterior mean ${\mu }_{* }$, and an uncertainty term, computed using the posterior variance ${\sigma }_{* }^{2}$.

#### Multi-fidelity modeling

The GPR framework, presented above, can be extended to construct probabilistic models able to consider numerous information sources of different fidelity levels^24^. Supposing that *s* levels of information source are available, the input, output data pairs can be organized by increasing fidelity as ${D}_{t}=\left\{{x}_{t},\,{y}_{t}\right\},{t}=1,\,2,\ldots ,{s}$. So, ${y}_{s}$ denotes the output of the most accurate and expensive to evaluate model, while ${y}_{1}$ denotes the output of the cheapest and least accurate model to evaluate. Assuming that only two models are available, a high-fidelity model and a low fidelity model, the high-fidelity model can be defined as a scaled sum of the low fidelity model plus an error term:

29 $${f}_{{high}}\left(x\right)=\rho {f}_{{low}}\left(x\right)+{f}_{{err}}\left(x\right)$$

where $\rho$ is a scaling constant quantifying the correlation between the two models and ${f}_{{err}}(x)$ denotes another GP which models the error.

A numerically efficient recursive inference scheme can then be constructed, by replacing the GP prior ${f}_{{low}}(x)$ with the GP posterior ${f}_{{lo}{w}_{{n}_{{low}}+1}}\left(x\right)$ of the previous inference level, while assuming that the corresponding experimental design sets {*D*_1_, *D*_2_, …, *D*_s_} have a nested structure. This implies that the training inputs of higher fidelity model needs to be a subset of the training inputs of the low fidelity model. This scheme is matching totally the Gaussian posterior distribution predicted by the fully coupled scheme, only now the inference problem is decoupled into two GPR problems, yielding the multi-fidelity posterior distribution $p\left({y}_{{high}},\,{X}_{{high}},{f}_{{lo}{w}_{{n}_{{low}}+1}}\right)$ with a predictive mean and variance at each level^18^.

30 $${\mu }_{{low}}({x}_{{n}_{{low}}+1})={\mu }_{{err}}+{k}_{{n}_{{low}}+1}{K}_{{low}}^{-1}\left[{y}_{{lo}{w}_{1:{n}_{{low}}}}-{\mu }_{{err}}\right]$$

31 $${\mu }_{{high}}({x}_{{n}_{{high}}})=\rho {\mu }_{{low}}\left({x}_{{n}_{{high}}+1}\right)+{\mu }_{{err}}+{k}_{{n}_{{high}+1}}{K}_{{high}}^{-1}\left[{y}_{{hig}{h}_{1:{n}_{{high}}}}-\rho {\mu }_{{low}}\left({x}_{{hig}{h}_{1:{n}_{{high}}}}\right)-{\mu }_{{err}}\right]$$

32 $${\sigma }_{{low}}^{2}\left({x}_{{n}_{{low}}+1}\right)=k({x}_{{n}_{{low}}+1},{x}_{{n}_{{low}}+1})-{k}_{{n}_{{low}}+1}\,{K}_{{low}}^{-1}\,{k}_{{n}_{{low}}+1}^{T}$$

33 $${\sigma }_{{high}}^{2}\left({x}_{{n}_{{high}}+1}\right)={\rho }^{2}{\sigma }_{{lo}{w}_{{n}_{{low}}+1}}^{2}\left({x}_{{n}_{{high}}+1}\right)+k\left({x}_{{n}_{{high}}+1},{x}_{{n}_{{high}}+1}\right)-{k}_{{n}_{{high}}+1}\,{K}_{{high}}^{-1}\,{k}_{{n}_{{high}}}^{T}$$

where ${n}_{{high}},\,{n}_{{low}}$ denote the number of training points from the high and low fidelity models, respectively.

#### Active learning

Let’s assume again that $n$ observations are available $\left\{{x}_{i},\,{y}_{i}\right\},{i}=1,\,\ldots ,{n}$ where ${y}_{i}=f\left({x}_{i}\right)$ and the next point to be evaluated $({x}_{n+1},\,{y}_{n+1})$ needs to be considered. The question that arises is if there is a more informed way to pick those points when evaluation is expensive to perform, rather than random picking.

This is achieved through an acquisition function $u(\cdot )$. The role of the acquisition function is to guide the search for the optimum. They are defined in a way such that high acquisition values correspond to a potential optimum of the unknown model $f$, large prediction uncertainty or a combination of those. Maximizing the acquisition function is used to select the next point to evaluate the function at. Consequently, the goal is to sample $f$ sequentially at ${argma}{x}_{x}u({x|D})$.

Every acquisition function depends on $\mu ,\,{\sigma }^{2}$ or a combination of both. The scale at which it depends on each one of those defines the exploration-exploitation tradeoff. When exploring, points where the GP variance is large should be chosen. When exploiting, points where the GP mean is closest to the extremum should be chosen. Many acquisition functions are available, some of them are presented in Table S4.

After sampling ${x}_{n+1}$ and evaluating ${f}_{n+1}$, GP regression is performed to fit to the new point as well. Then the process repeats itself until termination criteria are met, such as a maximum number of iterations, a minimum or maximum value is reached, or uncertainty is below an allowed value.

### Supplementary information

Supplementary Information

## Data Availability

The datasets generated during and/or analyzed during the current study are available in the Github repository, https://github.com/superlabs-gr/gpjet.

## Code availability

The source code of the GPJet framework is available to the readers through this public GitHub repository: https://github.com/superlabs-gr/gpjet.
