# Supplementary material for: Physics-Informed Bayesian learning of electrohydrodynamic polymer jet printing dynamics
Source: Commun Eng. 2023 Apr 29;2:20. doi: 10.1038/s44172-023-00069-0 (PMC10955830; doi:10.1038/s44172-023-00069-0)
Supplement: Supplementary file 1 — Supplementary Information [file 44172_2023_69_MOESM1_ESM.pdf]

Supplementary Notes

Video S1 and Video S2 published by Hrynevich et al.[1] was chosen as the source of the dataset used in this paper. A Sony Alpha 7 (Sony Corp. Japan) digital camera was used with a Nikon ED 200 mm lens (Nikon Corp. Japan). 1080 p resolution videos of the nozzle, jet and collector were taken at 50 frames per second. Process hyperparameters were set to 8 m s^{-2} and 500 m s^{-3} maximal stage acceleration and jerk, a 22G nozzle was used, polymer temperature was set to 87 ^{o}C and the voltage to the collector was set to -1.5kV, while the voltage to the nozzle was set to +5.75kV.

For Video S1 the air pressure feeding the nozzle was set to 1.2 bar and the distance between nozzle and collector was set to 3.5mm with a standard deviation 0.1mm. Collector’s speeds tested in Video S1 were 191.25 mm/min, 212.5 mm/min, 255 mm/min, 340 mm/min, 510 mm/min, 850 mm/min, 1530 mm/min and 2890 mm/min.

For Video S2 the air pressure feeding the nozzle was set to 2.4 bar and the distance between nozzle and collector was set to 4.5mm with a standard deviation 0.1mm. Collector's speeds tested in Video S2 were 292.5 mm/min, 520 mm/min, 1300 mm/min and 4420 mm/min.

Initially, the videos were split based on the collector speed setting. Then, the video frames were cropped to remove redundant pixels that would result to increased processing time. For real time video processing the user would need to specify the region of interest in the frame, so, as to crop and dispose needless information, as well as the position of the nozzle, the collector, and a factor, which represents the length of the Taylor cone depending on the nozzle's diameter.

Table S1: **Overview of curated dataset that emulates an E-jet printing machine.**

**Machine Setting**	**Air pressure** $p\ [bar]$	**Tip to Collector Distance** $Z\ [mm]$	**Collector Speed** $U_c\ [mm/min]$	**Number of frames**	**Duration** $[sec]$
1	1.2	3.5	191.2	1341	26.82
2	1.2	3.5	212.5	1672	33.44
3	1.2	3.5	255	1437	28.74
4	1.2	3.5	340	1343	26.86
5	1.2	3.5	510	648	12.96
6	1.2	3.5	850	613	12.26
7	1.2	3.5	1530	457	9.14
8	1.2	3.5	2890	401	8.02
9	2.4	4.5	292.5	1108	22.16
10	2.4	4.5	520	802	16.04

11	2.4	4.5	1300	812	16.24
12	2.4	4.5	4420	284	5.68

Table S2: Material properties of PCL

**Property**	**Value**
Zero-shear-rate (at 100℃) (η_o)	$1900\ Pa\ s$
Relaxation time (at 100℃) (λ_o)	$0.019\ s$
Activation energy of flow ($\Delta H/R_{ig}$)	$7938.4\ K$
Density (ρ)	$1145\ kg/m^3$
Heat capacity (C_p)	$1340\ J/kgK$
Thermal conductivity (k)	$0.14\ W/mK$
Electrical conductivity (K)	$9.5 * 10^{-9}\ S/m$
Surface tension (γ)	$0435\ N/m$
Ratio of solvent to zero-shear-rate viscosity (β)	0.001
Mobility factor (α)	0.015
Dielectric constant ratio ($\varepsilon/\varepsilon_o$)	2.9

Table S3: Typical values of dimensionless parameters used for PCL

**Parameter**	**Description**	**Value**
Bi	Biot number	0.424
De	Deborah number	1.14
Bo	Bond number	0
Re	Reynolds number	$5.785 * 10^{-6}$
Ca	Capillary number	1048.276
Na	Nahme-Griffith number	0.446
Fe	Electrostatic force number	0.0254
Γ	Temperature factor	21.283

Pe	Peclet number for thermal conductivity	105.209
Pe_c	Peclet number for electrical conductivity	0.1122

Table S4: Types of acquisition functions for Active Learning scheme

**Variance**	$\sigma^2(x)$	Purely exploration, makes sure, that we learn the function f everywhere on x to a similar level of absolute error.
**Probability of Improvement**	$PI(x) = \Phi\left(\frac{\mu(x) - f(x^+) - \xi}{\sigma(x)}\right)$, $\Phi(\cdot)$ is the normal cumulative distribution function	Selects the point most likely to offer an improvement of at least ξ but is extremely sensitive to the choice of the target.
**Expected Improvement**	$EI(x) = \{(\mu(x) - f(x^+) - \xi)\Phi(Z) + \sigma(x)\phi(Z),\ if\ \sigma(x) > 0 \quad 0,\ if\ \sigma(x) = 0$, where $Z = \{\frac{\mu(x)-f(x^+-\xi}{\sigma(x)},\ if\ \sigma(x) > 0 \quad 0 \quad ,\ if\ \sigma(x) = 0$, $\phi(\cdot)$ is the normal probability distribution function	Similar to PI but takes into account the magnitude of the improvement a point can potentially yield as well
**Lower Confidence Bound**	$LCB(x) = \mu(x) - \kappa\sigma(x)$, $\kappa > 0$	Selects points for evaluation based on the lower uncertainty bound

Table S5: Concentrated Nomenclature, containing every parameter's symbol, description, and units.

Parameters: Symbol – Description – Units		
**Process**		
R_0	Needle tip radius	mm
Z	Needle tip to collector distance	mm
t	Time	s

p	Pressure	kPa
Q	Volumetric flowrate	m^3/s
V_p	Voltage applied to needle tip	kV
V_c	Voltage applied to collector	kV
T_n	Needle temperature	℃
T_c	Collector temperature	℃
U_c	Collector speed	$mm\ s^{-1}$
U_{cr}	Critical collector speed	$mm\ s^{-1}$
**Jet**		
R	Jet radius	mm
V_j	Jet speed in y axis	$mm\ s^{-1}$
U_j	Jet speed in x axis	$mm\ s^{-1}$
V_{jm}	Jet speed on impact point	$mm\ s^{-1}$
θ_{jl}	Angle left	(°)
θ_{jr}	Angle right	(°)
A_j	Area	mm^2
L_j	Lag distance	mm
**Physics Jet Model**		
Re	Reynolds number	$-$
Pe	Peclet number	$-$
Ca	Capillary number	$-$
Fe	Electrostatic force parameter	$-$
Bo	Bond number	$-$
Na	Nahme-Griffith number	$-$
De	Deborah number	$-$
Bi_L	Local Biot number	$-$
Θ	Dimensionless temperature	$-$
Γ	Temperature factor	$-$
χ	Aspect ratio	$-$
α	Mobility factor	$-$
β	Ratio of solvent to zero-shear-rate viscosity	$-$
β_E	Dielectric constant ratio	$-$
σ	Surface charge density	C/m^2
E_T	The tangential component of the electric field to the jet surface	V/m
$f(\Theta)$	Temperature dependence of the zero-shear-rate viscosity	$-$
ΔH	Activation energy	K
R_{ig}	Ideal gas constant	$-$
ΔT_{Rh}	Temperature change necessary to substantially alter the rheological properties of the fluid	K
τ_{zz}	Total axial normal stress	Pa
τ_{rr}	Total radial normal stress	Pa

$\tau_{p,zz}$	Axial polymeric stress	Pa
$\tau_{p,rr}$	Radial polymeric stress	Pa
**Geometrical model**		
R_c	Steady coiling radius	mm
q	Jet's trace on the collector	mm
r	The contact point	mm
s	Deposited jet's arc length	mm
r	Polar radius coordinate	mm
ψ	Polar angle coordinate	(°)
θ	Curvature at the bottom of the jet	(°)
**CV Metrology**		
t_{CV}	Processing time	s
fps	Frames per second	s^{-1}
cf	Calibration factor	mm/pix
$stride$	Indicating every how many pixels along the z-axis we perform computations	pix
re	Jet's Right Edge at a specific point across the needle tip to collector distance (type: integer).	pix
le	Jet's Left Edge at a specific point across the needle tip to collector distance. (type: integer).	pix
$re_{previous}$	Jet's Right Edge at last accessed point before the currently processed point across the needle tip to collector distance (type: integer).	pix
$le_{previous}$	Jet's Right Edge at last accessed point before the currently processed point across the needle tip to collector distance (type: integer).	pix
$re_{current\_frame}$	Jet's Right Edge at current frame at all processed points across the needle tip to collector distance (type: array).	pix
$re_{previous\_frame}$	Jet's Right Edge at previous frame at all processed points across the needle tip to collector distance (type: array).	pix
**Gaussian Processes**		
D	Dataset, available input-output pair of observation data	−
$f(\cdot)$	Unknown function to be approximated	−
$m(\cdot)$	Mean function determining the unknown function	−
$K(\cdot)$	Covariance matrix determining the unknown function	−
$k(\cdot)$	Kernel reflecting the prior available knowledge on the unknown function	−

θ	Kernels hyperparameters to be trained	–
$\mu(\cdot)$	Mean prediction of GP model	–
$\sigma^2(\cdot)$	Variance prediction of GP model	–
n	Number of available data	–
**Multifidelity Modeling**		
$f_{high}(\cdot)$	High fidelity GP model	–
$f_{low}(\cdot)$	Low fidelity GP model	–
ρ	A scaling constant quantifying the correlation between the two models	–
$f_{err}(\cdot)$	Another GP modeling the bias term for the high-fidelity data	
n_{low}	Number of low-fidelity data available	–
n_{high}	Number of high-fidelity data available	–
**Active Learning and Bayesian Optimization**		
$u(\cdot)$	Acquisition function	–
ξ	Parameter specifying the least required improvement	–
$\Phi(\cdot)$	The normal cumulative distribution function	–
$\phi(\cdot)$	The normal probability distribution function	–
κ	Parameter specifying reliability of confidence intervals	–
**Performance Metrics**		
RMSE	The Root Mean Squared Error Performance Metric measures the average magnitude of the error between the predicted values and the true values.	-
MCIW	The Mean Confidence Interval Width Performance Metric measures the average width of a confidence interval.	-
Min. Regret	The Minimum Regret Performance Metric quantifies how well a method works at finding the optimum.	-

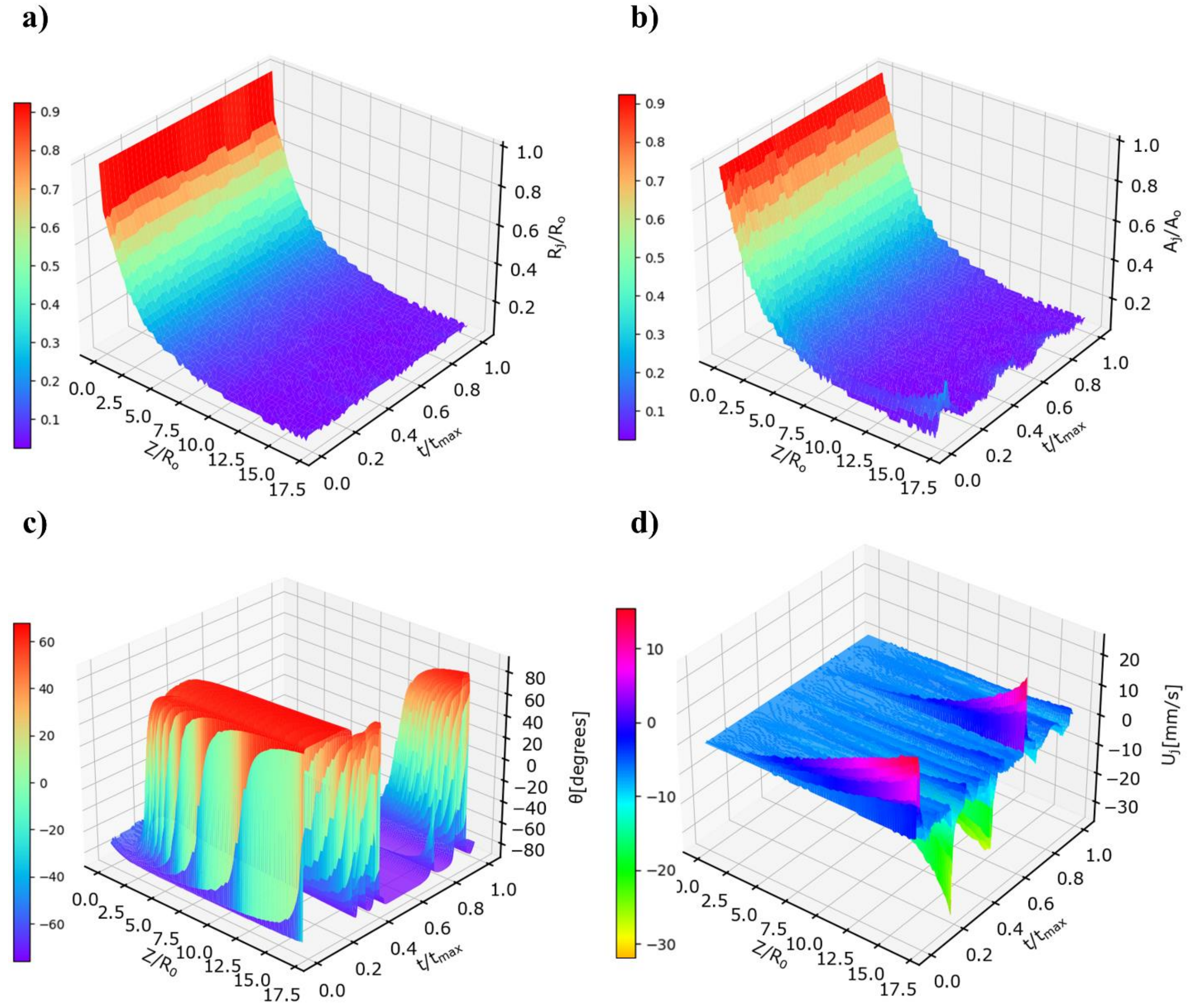

Figure S1: Features Extracted from Computer Vision Module. a) Normalized jet radius (R_j/R_o) obtained from the computer vision metrology module of the GPJet framework plotted against the normalized jet length (Z/R_o) and the normalized time (t/t_{max}). b) Normalized jet area (A_j/A_o) obtained from the computer vision metrology module of the GPJet framework plotted against the normalized jet length (Z/R_o) and the normalized time (t/t_{max}). c) Jet angles (θ) obtained from the computer vision metrology module of the GPJet framework plotted against the normalized jet length (Z/R_o) and the normalized time (t/t_{max}). d) Jet velocities (U_j) obtained from the computer vision metrology module of the GPJet framework plotted against the normalized jet length (Z/R_o) and the normalized time (t/t_{max}).

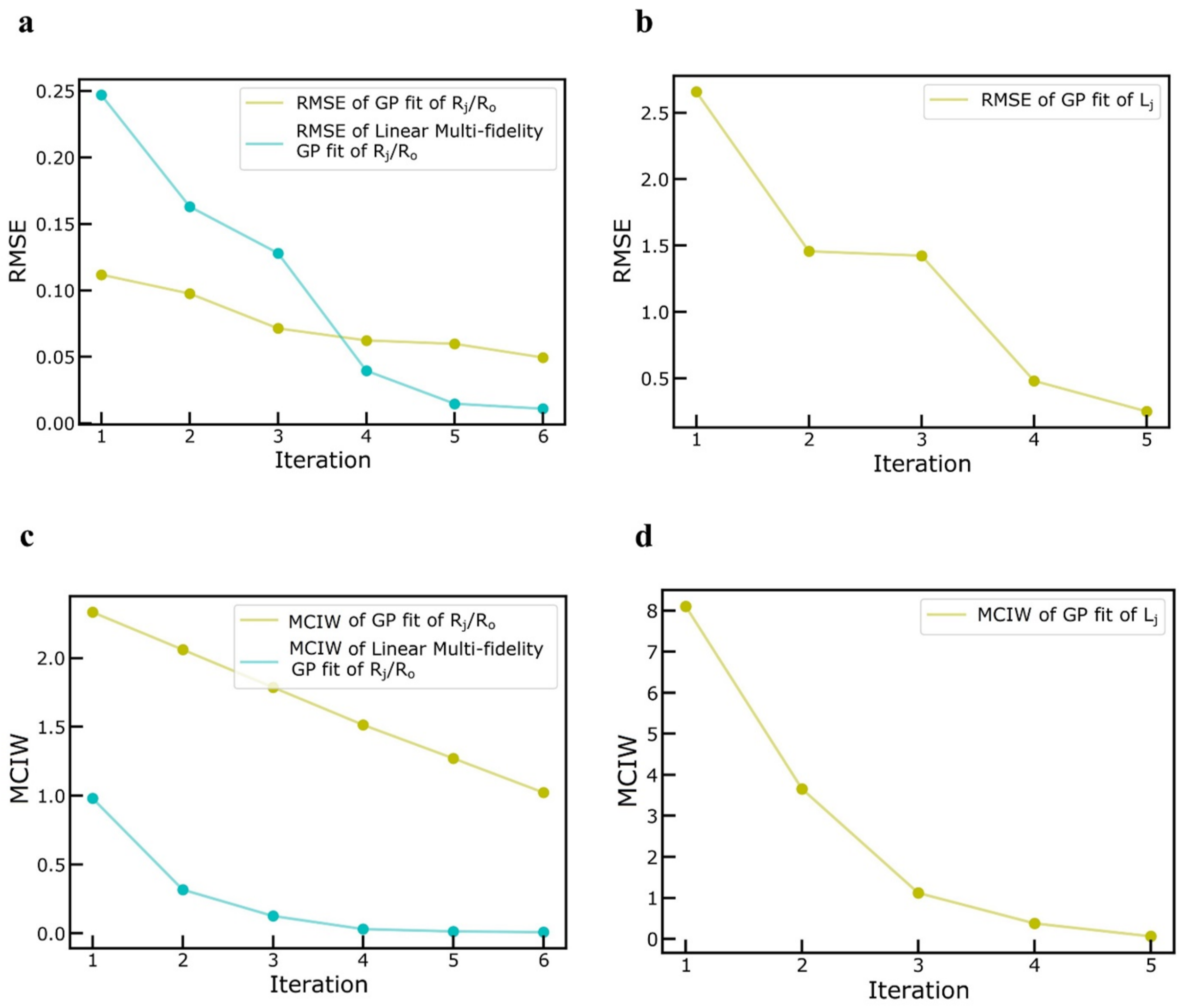

Figure S2: Performance Metrics Evolution for Active Learning Tasks presented in Figure 7 and Figure 8. a) Root Mean Squared Error (RMSE) evolution after each iteration, regarding the normalized jet radius (R_j/R_o). **b)** Root Mean Squared Error (RMSE) evolution after each iteration, regarding the lag distance (L_j). **c)** Mean Confidence Interval Width (MCIW) evolution after each iteration, regarding the normalized jet radius (R_j/R_o). **d)** Mean Confidence Interval Width (MCIW) evolution after each iteration, regarding the lag distance (L_j).

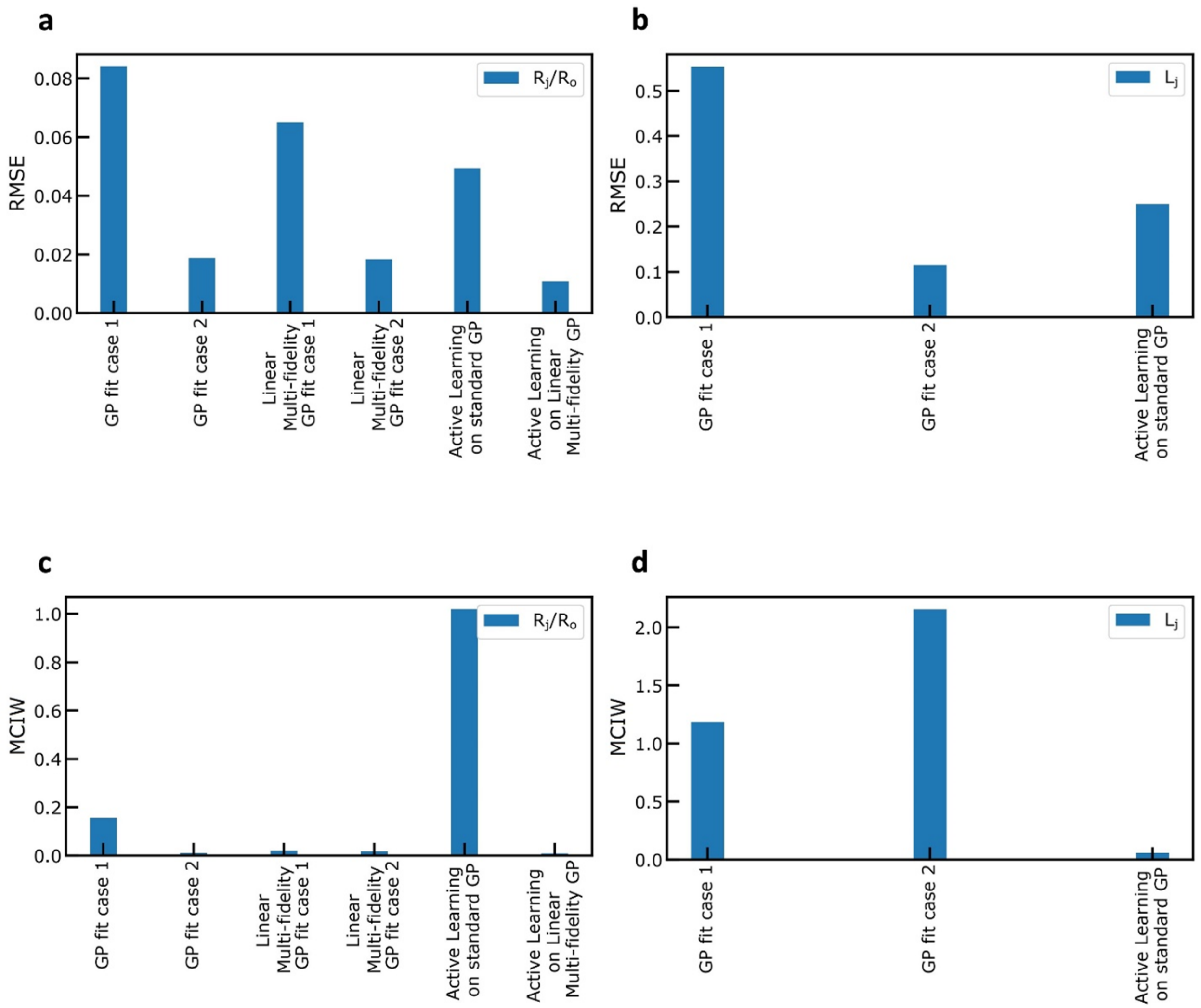

Figure S3: Collective Performance Metrics for Regression and Active Learning Tasks. a) Root Mean Squared Error (RMSE) for every case, regarding the normalized jet radius (R_j/R_o). **b)** Root Mean Squared Error (RMSE) performance metric, for every case, regarding the lag distance (L_j). **c)** Mean Confidence Interval Width (MCIW) performance metric, for every case, regarding the normalized jet radius (R_j/R_o). **d)** Mean Confidence Interval Width (MCIW) performance metric, for every case, regarding the lag distance (L_j).

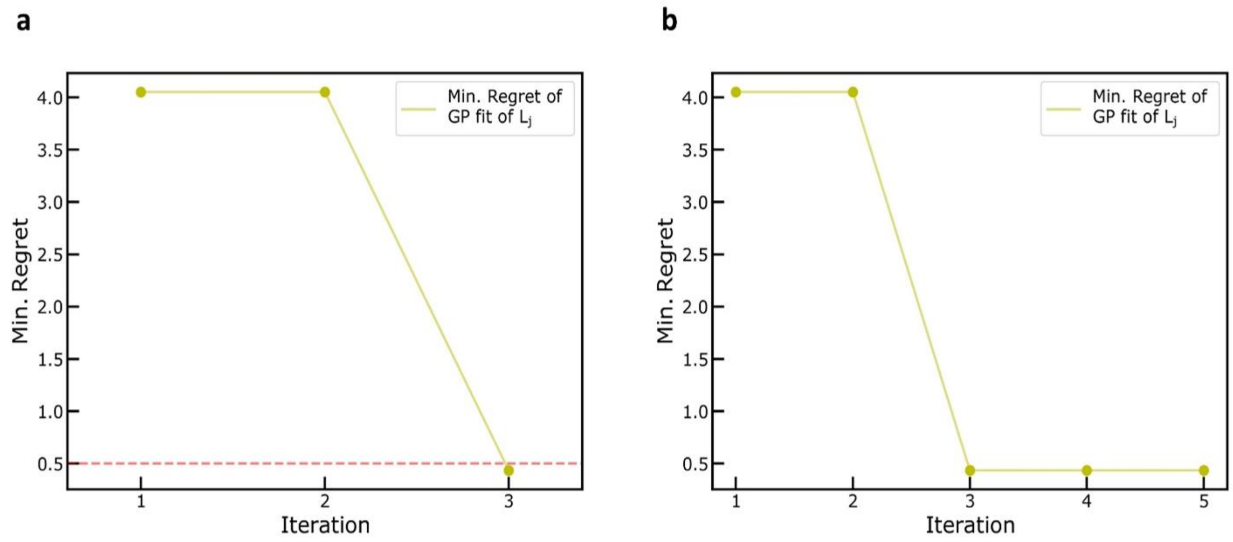

Figure S4: Performance Metrics. a) Minimum Regret Performance Metric evolution, after each iteration, regarding the Bayesian Optimization Task to find the minimum lag-distance (L_j). The termination criterion is set to 0.5 mm (red dashed line). **b)** Minimum Regret Performance Metric evolution, after each iteration, regarding the Active Learning Task to explore the design space of lag-distance (L_j) for specific speed ratios (U_c/V_{jm}).

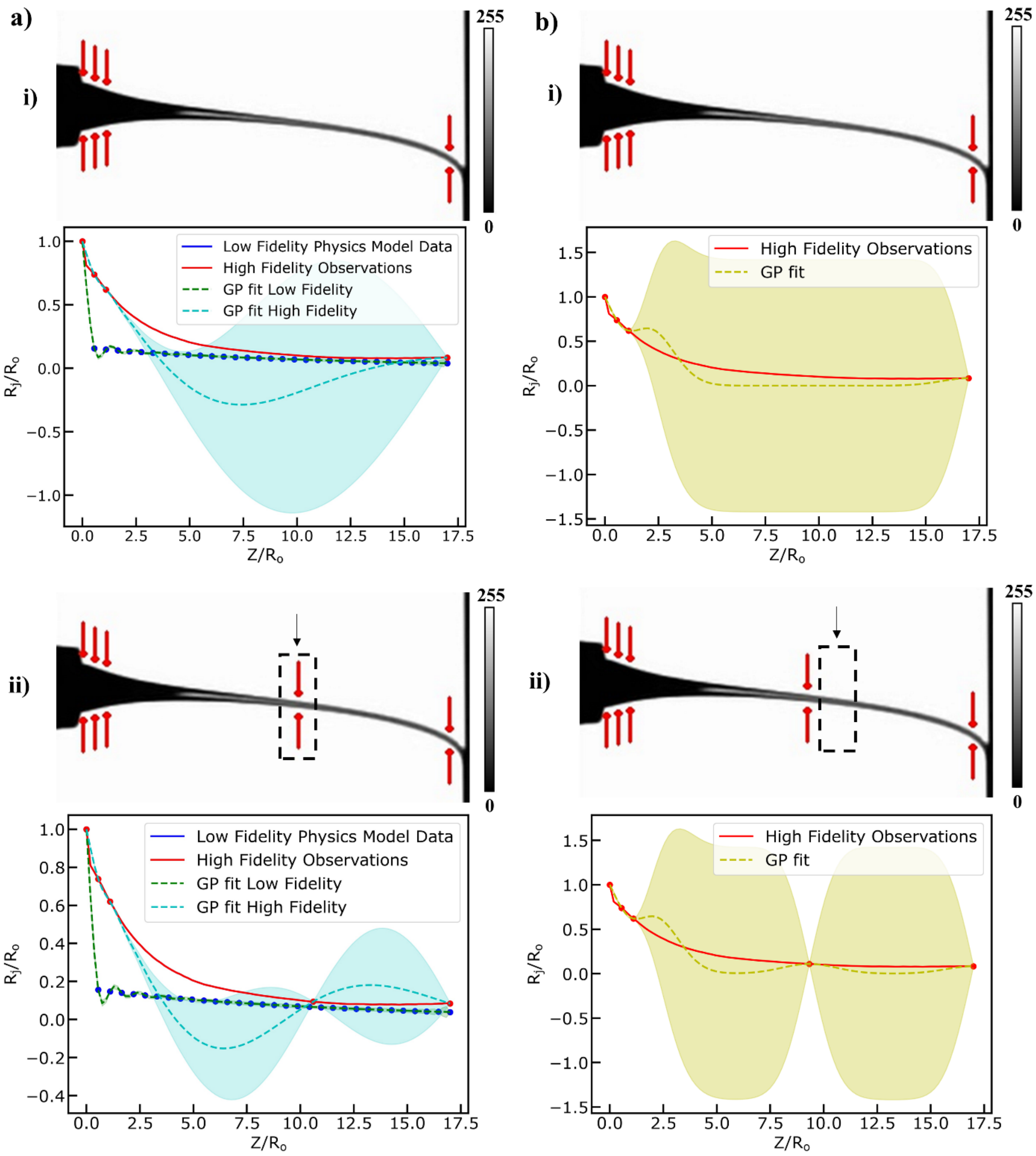

a)
b)
i)
ii)
255
0
Low Fidelity Physics Model Data
High Fidelity Observations
GP fit Low Fidelity
GP fit High Fidelity
GP fit
R_j/R_o
Z/R_o

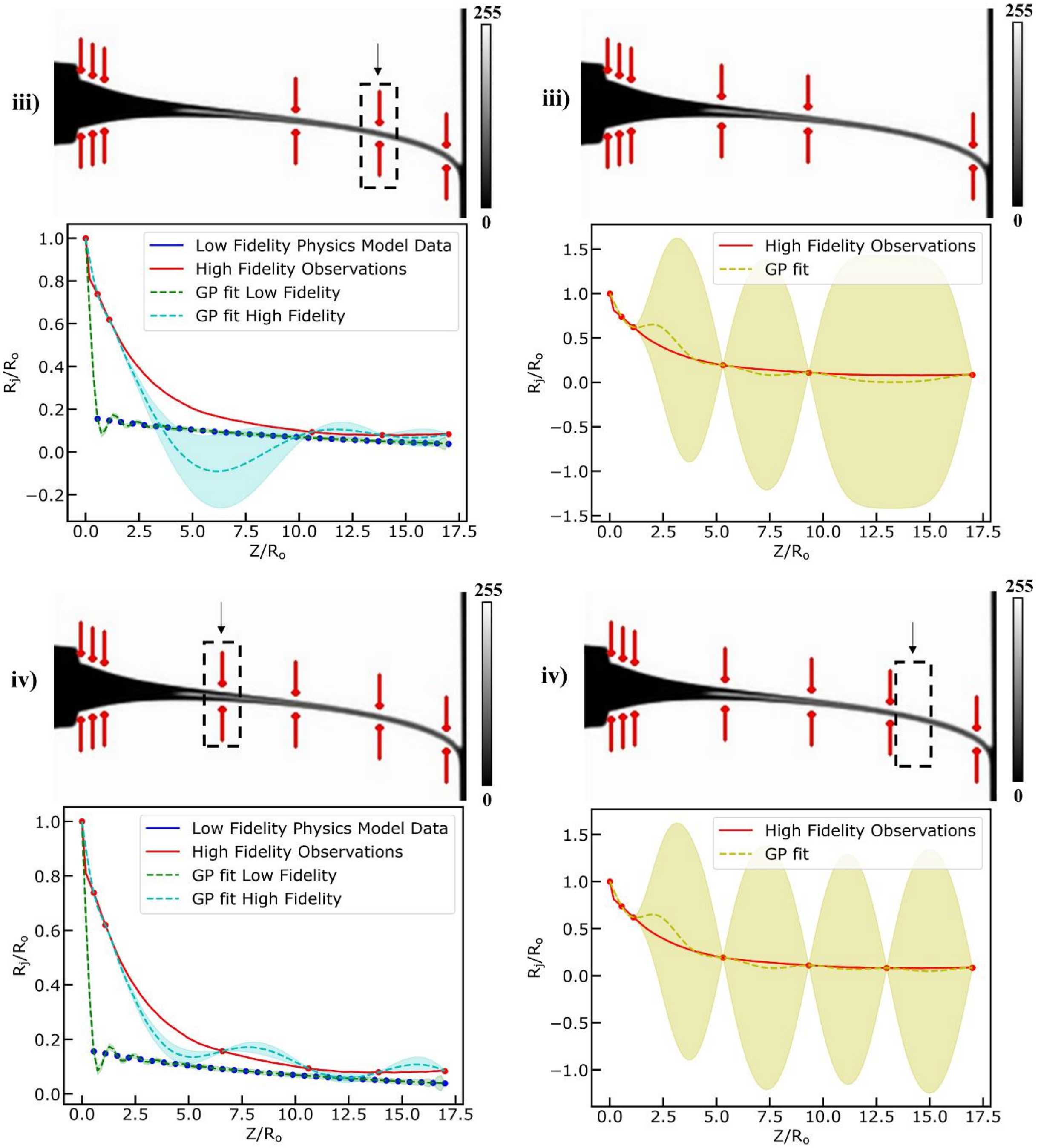

iii)
iii)
iv)
iv)
255
0
Low Fidelity Physics Model Data
High Fidelity Observations
GP fit Low Fidelity
GP fit High Fidelity
GP fit
R_j/R_o
Z/R_o

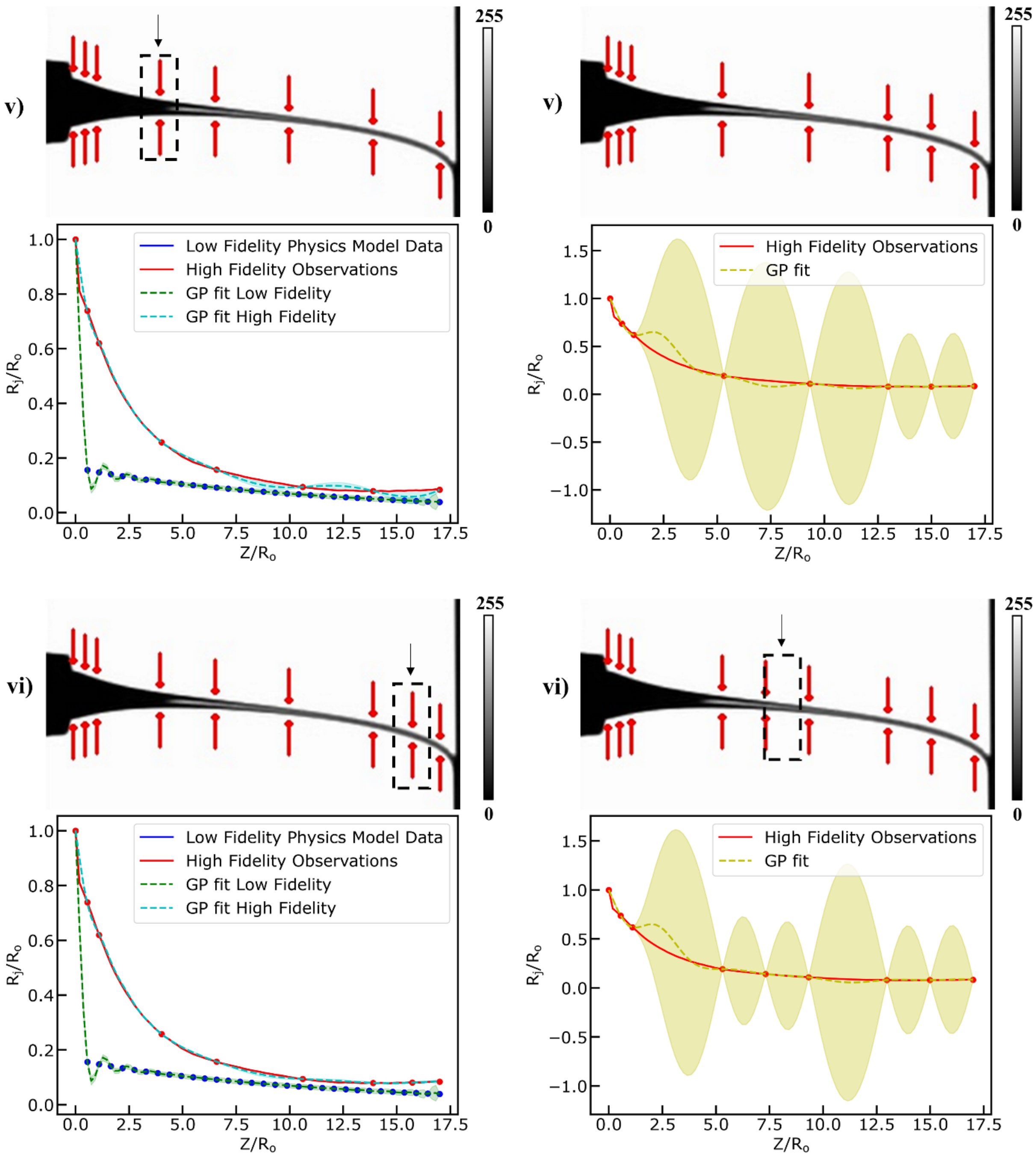

Figure S5: Results of Active Learning process on Multifidelity Modeling Task (column a) versus on High Fidelity Modeling Task (column b). a) exploring the design space using Active Learning to fit a Multifidelity Gaussian Process to normalized high fidelity observation data (red color) of jet radius (R_j/R_o) and low fidelity model data obtained from the computer vision metrology module of the GPJet framework and from the multi-physics model, respectively, at specific z axis coordinates along the normalized jet length (Z/R_o), (**i – vi**) denote the iterations of the active learning algorithm until it meets its termination criteria). **b**) exploring the design space using Active Learning to fit a Gaussian Process to normalized high fidelity observation data (red color) of jet radius (R_j/R_o) obtained from the computer vision metrology module of the GPJet framework at specific z axis coordinates along the normalized jet length (Z/R_o), (**i – vi**) denote the iterations of the active learning algorithm until it meets its termination criteria). In every case, the observation point chosen at each iteration is denoted with a black dashed line box pointed by a black arrow.

Supplementary References

1. Hrynevich, A., Liashenko, I. & Dalton, P. D. Accurate Prediction of Melt Electrowritten Laydown Patterns from Simple Geometrical Considerations. *Adv Mater Technologies* 5, 2000772 (2020).
